# Sex differences in nicotine intake and relapse behavior in nicotine-dependent adult wistar rats

**DOI:** 10.3389/fphar.2024.1415219

**Published:** 2024-09-26

**Authors:** Ranjithkumar Chellian, Azin Behnood-Rod, Adriaan W. Bruijnzeel

**Affiliations:** Department of Psychiatry, University of Florida, Gainesville, FL, United States

**Keywords:** smoking, nicotine, self-administration, sex, dependence, withdrawal, drug seeking

## Abstract

**Introduction:**

Tobacco use is highly addictive and the leading cause of premature mortality in the world. Long-access nicotine self-administration procedures in rats closely model human smoking behavior. However, significant gaps remain in our understanding of sex differences in the development of dependence and relapse in adult rats.

**Methods:**

In the present study, we investigated operant responding for both nicotine and saline and the development of dependence in adult rats of both sexes. The rats had daily access to nicotine or saline for 6 h per day, 7 days per week. Dependence was assessed by evaluating precipitated and spontaneous somatic withdrawal signs, measuring locomotor activity in the small open field test, and assessing anxiety-like behavior in the large open field and elevated plus maze test. The sucrose preference test was used to determine if cessation of nicotine intake leads to anhedonia. It was also investigated if a period of forced abstinence affects nicotine-seeking behavior.

**Results:**

This study showed that nicotine intake is higher in females than in males when given daily long access to nicotine. Daily nicotine self-administration led to more precipitated and spontaneous somatic withdrawal signs compared to saline self-administration, with no sex differences observed. In addition, cessation of nicotine intake led to a similar increase in activity in both males and females in the small open field test. However, cessation of nicotine intake did not increase anxiety-like behavior or cause anhedonia in either males or females. A time course analysis revealed that the nicotinic acetylcholine receptor antagonist mecamylamine affected nicotine intake differently in males and females, increasing intake in males and decreasing intake in females. Three weeks of forced abstinence led to an increase in nicotine and saline-seeking behavior. The rats exhibited more nicotine than saline seeking, and the females displayed more nicotine seeking than the males.

**Discussion:**

The present findings demonstrate that females self-administer more nicotine and display more nicotine-seeking behavior than males. Furthermore, there were no sex differences in somatic withdrawal signs or activity during abstinence from nicotine. This work underscores the importance of considering sex differences across various aspects of addiction, including intake and relapse, when developing novel treatments for tobacco use disorder.

## 1 Introduction

Tobacco use disorder is characterized by a loss of control over smoking, a strong desire to smoke, craving for cigarettes, and withdrawal signs upon a smoking cessation attempt (American Psychiatric Association, 2013). Worldwide, there are about 1.3 billion people who use tobacco products, and eighty percent of them live in low- and middle-income countries (WHO, 2021). Smoking is the leading preventable cause of disease and death in the world. Smoking increases the risk for a wide range of diseases, including chronic obstructive pulmonary disease, cardiovascular disease, cancer, and Alzheimer’s disease (Ott et al., 1998; Ambrose and Barua, 2004; Sasco et al., 2004; Forey et al., 2011). Furthermore, smoking induces changes in the brain that increase the risk for psychiatric disorders, including depression (Bruijnzeel et al., 2011; Bruijnzeel, 2012a). Although the use of electronic cigarettes is on the rise, smoking remains the primary method of nicotine consumption (Cornelius et al., 2020). There is an urgent need for new animal models that allow the evaluation of potential smoking cessation treatments to develop novel therapies for tobacco-use disorder. Because in many countries, more than thirty percent of smokers are female, it is imperative to evaluate whether there are sex differences in nicotine intake, the development of dependence, and relapse in animal models of tobacco use disorder (Statista, 2021).

Nicotine is highly addictive and the primary psychoactive compound in tobacco and e-liquid that sustains smoking and vaping (Stolerman and Jarvis, 1995; Kinnunen et al., 2019). Animal models have been developed to study both the positive and negative reinforcing properties of nicotine. Nicotine induces mild euphoria and cognitive enhancement, which significantly contributes to the initiation and maintenance of smoking (Wesnes and Warburton, 1983; Pomerleau and Pomerleau, 1992). The negative reinforcing properties of nicotine, including anhedonia, anxiety, and craving, also play a pivotal role in the maintenance of smoking and contribute to relapse following abstinence (Bruijnzeel, 2012b). The methods for intravenous nicotine self-administration in rats were established during the late 1970s (Lang et al., 1977; Hanson et al., 1979). Some of the first studies showed that blockade of nicotinic acetylcholine receptors (nAChRs) and dopamine D1 receptors decreases the reinforcing properties of nicotine in rats with short access (1 h/day) to nicotine (Corrigall and Coen, 1989; 1991). Blockade of nAChRs and dopamine D1 receptors also prevents the development of nicotine-induced place preference in rats (Fudala et al., 1985; Acquas et al., 1989). Furthermore, the United States Food and Drug Administration (FDA)-approved smoking cessation drugs bupropion and varenicline decrease nicotine intake in rats with short access to nicotine (Bruijnzeel and Markou, 2003; O'Connor et al., 2010). The negative reinforcing properties of nicotine have been mainly investigated in rats that received nicotine noncontingently via minipumps. Cessation of noncontingent nicotine administration leads to somatic withdrawal signs, anhedonia, hyperalgesia, and cognitive impairments (Shoaib and Bizarro, 2005; Rylkova et al., 2008; Bruijnzeel et al., 2009; Yohn et al., 2014; Bagdas et al., 2018). Treatment with bupropion and varenicline diminishes somatic and affective withdrawal signs associated with the cessation of noncontingent nicotine administration (Cryan et al., 2003; Igari et al., 2013).

Adaptations induced by drug self-administration may more closely align with those observed in drug users than those induced by noncontingent drug administration (Markou et al., 1999; Jacobs et al., 2003; Kuhn et al., 2019; Schweppe et al., 2020). Therefore, there is growing interest in studying the development of dependence in animals that self-administer nicotine. Paterson and Markou compared the development of dependence in rats with short (1 h/day, 5 and 7 days/week) and long (6 h/day, 7 days/week) access to nicotine (Paterson and Markou, 2004). This study showed that animals with daily access to nicotine (1 and 6 h) developed dependence but not those that self-administered nicotine only 5 days per week. On a similar note, the nAChRs antagonist mecamylamine precipitated somatic withdrawal signs in rats with daily access but not in those that did not have daily access to nicotine. Thus, daily nicotine intake is critical for the development of dependence in rodents. The dose of nicotine also plays a role in the development of nicotine dependence. O'Dell et al. (2007) demonstrated that, in rats with daily long access to nicotine, those self-administering 0.06 mg/kg/inf of nicotine had higher levels of nicotine intake and displayed more mecamylamine-precipitated withdrawal signs compared to those self-administering 0.015 mg/kg/inf of nicotine. It is unlikely that doses higher than 0.06 mg/kg/inf lead to more severe dependence in rats, as nicotine intake only slightly increases when the dose is increased above 0.06 mg/kg/inf (Donny E. et al., 2000; O'Dell and Koob, 2007). These findings indicate that rats are most likely to develop dependence when they have daily access to nicotine and self-administer the 0.06 mg/kg/inf dose or slightly higher doses.

Previous studies demonstrated that daily access to nicotine leads to dependence in adult male rats (for a review on this topic, see (Chellian et al., 2022a)). However, there remain many gaps in our understanding of the development of dependence and relapse in adult animals that self-administer nicotine. For example, it is not known whether there are sex differences in nicotine intake in animals with long access to nicotine, and if the development of nicotine dependence follows the same trajectory in male and female rats. Furthermore, important control groups that self-administered saline were not included in prior studies. Therefore, in the present study, we investigated operant responding for both nicotine and saline and the development of dependence in adult male and female rats. The rats had daily access to nicotine or saline for 6 h per day, 7 days per week. The development of dependence was investigated by assessing precipitated and spontaneous somatic withdrawal signs and measuring anxiety-like behavior in the large open field and elevated plus maze test (Rylkova et al., 2008; Bauzo and Bruijnzeel, 2012; Knight et al., 2021). Furthermore, the sucrose preference test was conducted to determine if cessation of nicotine intake leads to anhedonia (Primo et al., 2023). At the end of the study, it was investigated if a period of forced abstinence affects nicotine and saline seeking behavior. The present study showed that nicotine intake and nicotine seeking is higher in females than males with daily long access to nicotine. Furthermore, nicotine self-administration led to the development of dependence, as indicated by somatic withdrawal signs, but no sex differences were observed. In addition, cessation of nicotine intake increased activity in the small open field test, but it did not lead to an increase in anxiety-like behavior or anhedonia in the males and the females.

## 2 Materials and methods

### 2.1 Animals

Adult male (200–250 g, 8–9 weeks of age; N = 28) and female (175–225 g, 8–9 weeks of age; N = 28) Wistar rats were purchased from Charles River (Raleigh, NC). The rats were housed with a rat of the same sex in a climate-controlled vivarium on a reversed 12 h light-dark cycle (light off at 7 a.m.). The rats were handled for 2–3 min per day for several days before the food training sessions. During the food training period, the rats were singly housed and remained singly housed for the rest of the study. Prior to the onset of the studies, food was available *ad libitum* in the home cage. During the food training, and the nicotine and saline self-administration sessions, the rats were fed 90–95 percent of their *ad libitum* food intake (males: 23 g, females: 19 g). A mild level of food restriction facilitates food training and nicotine self-administration in rats (Donny et al., 1998; Garcia et al., 2014). Water was available *ad libitum* throughout the study except for 1 day when the rats had only access to two bottles with a 2% w/v sucrose solution (see sucrose preference test for details). The experimental protocols were approved by the University of Florida Institutional Animal Care and Use Committee (IACUC). All experiments were performed in accordance with relevant IACUC guidelines and regulations and in compliance with ARRIVE guidelines 2.0 (Animal Research: Reporting of *In Vivo* Experiments).

### 2.2 Drugs

For intravenous self-administration, (−)-nicotine hydrogen tartrate (NIDA Drug Supply Program) was dissolved in sterile saline (0.9% sodium chloride), and the pH was adjusted to 7.2 ± 0.2 using 1 M NaOH. The rats self-administered 0.03 or 0.06 mg/kg/inf of nicotine in a volume of 0.1 mL/inf. Nicotine doses are expressed as base. For drug treatments, (−)-nicotine hydrogen tartrate and mecamylamine hydrochloride (NIDA Drug Supply Program) were dissolved in sterile saline and administered subcutaneously (SC) in a volume of 1 mL/kg body weight. The nicotine dose is expressed as base and the mecamylamine dose is expressed as salt.

### 2.3 Experimental design

**FIGURE 1:**
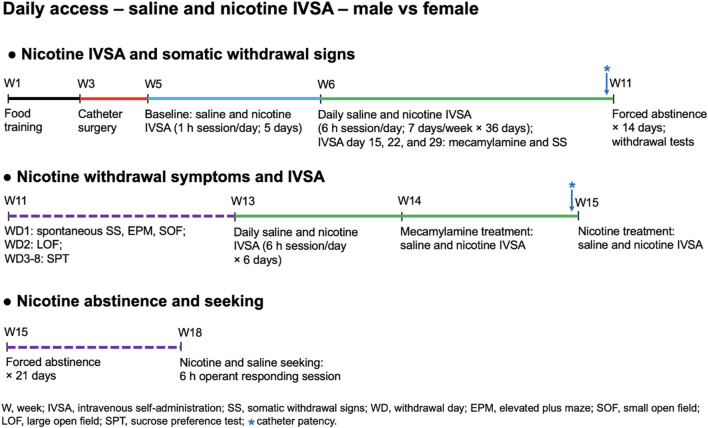
Schematic overview of the experiment. Adult male and female Wistar rats were trained to respond for food pellets and were then prepared with IV catheters. The rats were allowed to self-administer saline or nicotine in five 1-h baseline sessions and then the rats self-administer saline or nicotine in 6-h sessions/day for 36 days. Mecamylamine-precipitated somatic withdrawal signs were assessed after the 15th, 22nd, and 29th nicotine and saline self-administration sessions. Spontaneous somatic withdrawal signs, locomotor activity, and affective withdrawal symptoms (anxiety and anhedonia) were determined during forced abstinence period. Fourteen days after nicotine withdrawal, the effects of forced abstinence on nicotine and saline self-administration were investigated. After six nicotine and saline self-administration sessions, the effects of mecamylamine and nicotine treatment on nicotine and saline self-administration were investigated. After completing the last nicotine and saline self-administration sessions, the rats were housed in the vivarium for 21 days and the effects of forced abstinence on nicotine and saline seeking behavior were investigated.

All rats in this study underwent identical procedures and successive tests, with the only difference being that the control groups self-administered saline, while the experimental groups self-administered nicotine. A schematic overview of the experimental design is depicted in Figure 1. Before the catheter surgeries, rats underwent 10 days of food training. After 5 days of food training, the rats were singly housed and remained so for the duration of the study. Upon completion of the training, the rats were prepared with catheters in the jugular vein. The catheter surgery was followed by a 7-day recovery period. The male (N = 28) and female (N = 28) rats were each divided into two self-administration groups: nicotine (N = 14/sex) and saline (N = 14/sex). The rats initially self-administered nicotine or saline for five 1-h baseline sessions. This was followed by 36 days of daily 6-h long access self-administration sessions. To investigate the development of dependence during this period, precipitated somatic withdrawal signs were examined by administering mecamylamine after the 15th, 22nd, and 29th self-administration session. After thirty-six self-administration sessions, there was a 14-day forced abstinence period. To further investigate the development of dependence, spontaneous withdrawal was examined during this forced abstinence period. Spontaneous somatic withdrawal signs were recorded 16–17 h after the last self-administration session. Immediately after counting somatic signs, anxiety-like behavior was measured in the elevated plus-maze test, and then locomotor activity was assessed in the small open field test. Furthermore, 48 h after the last nicotine and saline self-administration session, anxiety-like behavior was measured in the large open field test. To determine if the cessation of nicotine self-administration leads to anhedonia, the sucrose preference test was conducted from withdrawal day 3 to day 8. Subsequently, it was investigated whether a 14-day forced abstinence period affects nicotine and saline intake. After the abstinence period, the rats were allowed to self-administer nicotine and saline for 6 days. After these six self-administration sessions, the effects of mecamylamine and nicotine treatment on nicotine and saline self-administration was investigated. This was followed by a 21-day forced abstinence period, after which nicotine and saline-seeking behavior was investigated. Nicotine and saline-seeking behavior were investigated during a 6-h session. The rats were fed 90–95 percent of their *ad libitum* food intake during food training (Weeks 1–3, Figure 1), the first self-administration period (Weeks 5–11), and the second self-administration period (Weeks 13–15). The rats were fed *ad libitum* before the onset of food training, during the period when the catheter surgeries were conducted (Weeks 3–5), and during the first (Weeks 11–13) and second (Weeks 15–18) forced abstinence period.

### 2.4 Food training

Rats were trained to press a lever for food pellets in operant chambers that were placed in sound- and light-attenuated cubicles (Med Associates, St. Albans, VT). Responding on the active lever resulted in the delivery of a food pellet (45 mg, F0299, Bio-Serv, Frenchtown, NJ), and responding on the inactive lever was recorded but did not have scheduled consequences. Food delivery was paired with a cue light, which remained illuminated throughout the time-out (TO) period. The food training sessions were conducted for 10 days. Instrumental training started under a fixed ratio 1 (FR1) time-out 1 s (TO1s) reinforcement schedule, and the rats remained on this schedule for 5 days (30 min sessions per day). After the fifth food training session, the rats were singly housed and remained so for the rest of the study. On day 6, the time-out period was increased to 10 s. The rats were allowed to respond for food pellets under the FR1-TO10s schedule (20 min sessions) for 5 days. Both levers were retracted during the 10 s time-out period.

### 2.5 Intravenous catheter implantation

The catheters were implanted as described before (Chellian et al., 2021b; Chellian et al., 2021c; Chellian et al., 2022b). The rats were anesthetized with an isoflurane-oxygen vapor mixture (1%–3%) and prepared with a catheter in the right jugular vein. The catheters consisted of polyurethane tubing (length 10 cm, inner diameter 0.64 mm, outer diameter 1.0 mm, model 3Fr, Instech Laboratories, Plymouth Meeting, PA). The right jugular vein was isolated, and the catheter was inserted 2.9 cm for males and 2.5 cm for females. The tubing was then tunneled subcutaneously and connected to a vascular access button (Instech Laboratories, Plymouth Meeting, PA). The button was exteriorized through a 1-cm incision between the scapulae. During the 7-day recovery period, the rats received daily infusions of the antibiotic Gentamycin (4 mg/kg, IV, Sigma-Aldrich, St. Louis, MO). A sterile heparin solution (0.1 mL, 50 U/mL) was flushed through the catheter before and after administering the antibiotic and after nicotine self-administration. After flushing the catheter, 0.05 mL of a sterile heparin/glycerol lock solution (500 U/mL, Instech Laboratories, Plymouth Meeting, PA) was infused into the catheter. The animals received carprofen (5 mg/kg, SC) daily for 72 h after the surgery. Three days before the start of the nicotine self-administration sessions, the rats were allowed to respond for food pellets under the FR1-TO10s schedule (one 20-min session). Catheter patency was evaluated with Brevital at the end of the first and second self-administration period (Figure 1). The catheters were tested by infusing 0.2 mL of the ultra-short-acting barbiturate Brevital (1% methohexital sodium). Rats with patent catheters displayed a sudden loss of muscle tone. If the rats did not respond to Brevital, their self-administration data were excluded from the analysis. Six rats (2 male nicotine, 2 female saline, and 2 female nicotine rats) did not respond to Brevital during the first test, and therefore, their data were not included in the study. Four rats (1 male saline, 2 male nicotine, and 1 female nicotine) did not respond to Brevital during the second test and their data were excluded after week 12 (Figure 1).

### 2.6 Baseline and thirty-six daily nicotine and saline self-administration sessions

Male (N = 28) and female (N = 28) rats were each divided into two self-administration groups: nicotine (N = 14/sex) and saline (N = 14/sex). Animals in the control groups self-administered saline and all self-administration procedures were the same for the saline groups and the nicotine groups. Briefly, the rats were allowed to self-administer nicotine and saline for five daily 1-h baseline sessions. During the first three sessions (days 1–3), the rats self-administered saline and 0.03 mg/kg/inf of nicotine under an FR1-TO10s schedule. During the following two sessions (days 4 and 5) the rats self-administered saline and 0.06 mg/kg/inf of nicotine under an FR1-TO60s schedule. Rats that self-administer 0.06 mg/kg/inf of nicotine have a higher level of nicotine intake and more somatic withdrawal signs compared to rats that self-administered 0.03 mg/kg/inf of nicotine (Shoaib and Stolerman, 1999; Chaudhri et al., 2005). High doses of nicotine can cause seizures in rodents (Hanson, 1979; Corrigall and Coen, 1989; Damaj et al., 1999). To prevent seizures, the time-out period was increased from 10 to 60 s when the dose of nicotine was increased from 0.03 to 0.06 mg/kg/inf. Total nicotine intake over a 1-h nicotine self-administration period is not affected by the time-out period (10–60 s)(Corrigall and Coen, 1989). During the first day that the rats received the 0.03 or 0.06 mg/kg/inf dose, nicotine intake was limited to prevent aversive effects (i.e., seizures and dysphoria). The maximum number of infusions was set to 20 on the first day that the rats received the 0.03 mg/kg/inf of nicotine dose and to 10 on the first day that the rats received the 0.06 mg/kg/inf dose. On these days, saline infusions were limited to a similar degree as the nicotine infusions. After five self-administration sessions, rats in the saline group continued to self-administer saline, and rats in the nicotine group continued to self-administer nicotine (0.06 mg/kg/inf) daily for 36 days under an FR1-TO60s schedule in 6-h sessions. Responding on the active lever resulted in the delivery of a nicotine or a saline infusion (0.1 mL infused over a 6.5-s period). Active lever presses include both effective active lever presses, which turn on the pump and cue light, and ineffective active lever presses, which have no scheduled consequences (Kosten et al., 2004; Flagel et al., 2010). The ineffective active lever presses occur immediately after an active lever press and before the lever has been fully retracted. The initiation of the delivery of an infusion was paired with a cue light, which remained illuminated throughout the time-out period. Responding on the inactive lever was recorded but did not have scheduled consequences. The active and inactive levers were retracted during the time-out period. During the 6-h nicotine and saline self-administration sessions, the rats had access to water in the operant chambers. On day 36, the rats received *ad libitum* food in their home cage after completing the self-administration sessions.

### 2.7 Mecamylamine-precipitated somatic withdrawal signs

Somatic signs were observed in a transparent Plexiglas observation chamber (25 cm × 25 cm x 46 cm) with 1 cm of corncob bedding as described previously (Rylkova et al., 2008; Yamada et al., 2010). The rats were habituated to the observation chambers for 5 min per day on 3 consecutive days before the somatic withdrawal test. The rats received mecamylamine injections (2 mg/kg, SC) immediately after the 15th, 22nd, and 29th nicotine and saline self-administration sessions. Ten minutes after the mecamylamine injections the rats were placed in the observation chamber and somatic withdrawal signs were recorded for 10 min. The following somatic withdrawal signs were recorded: body shakes, head shakes, chews, teeth chattering, cheek tremors, gasps, writhes, ptosis, genital licks, foot licks, and yawns. Ptosis was counted once per minute if present continuously. Somatic signs were observed in a quiet, brightly lit room. The total number of somatic signs was the sum of the individual occurrences.

### 2.8 Spontaneous nicotine withdrawal

#### 2.8.1 Withdrawal day 1: spontaneous somatic withdrawal signs, elevated plus maze test, and small open field test

Spontaneous somatic withdrawal signs were recorded 16–17 h after the last nicotine and saline self-administration session. Somatic signs were observed for 20 min in a transparent Plexiglas observation chamber (Rylkova et al., 2008; Yamada et al., 2010). After recording the somatic withdrawal signs, anxiety-like behavior was measured in the elevated plus-maze test for 5 min. The elevated plus-maze test is used to measure anxiety-like behavior in rodents and was performed as described in our previous work (Knight et al., 2021; Bruijnzeel et al., 2022). The elevated plus maze apparatus (Coulbourn Instruments, Holliston, MA) consisted of two closed arms (i.e., with black walls, 50 cm × 10 cm × 30 cm; Length × Width × Height, L × W × H) and two open arms (i.e., without walls; 50 cm × 10 cm; L × W). The open and closed arms were connected by a central platform, and the open arms had 0.5 cm tall ledges to prevent the rats from falling off. The open arms were placed opposite of each other, and the maze was elevated 55 cm above the floor on acrylic legs. At the beginning of each test, the rats were placed in the central area facing an open arm and were allowed to explore the apparatus for 5 min. The rats were recorded with a camera mounted above the maze, and the test was conducted in a quiet, dimly lit room (100 lux). The open-arm and closed-arm duration, the number of open and closed-arm entries, and total distance traveled were determined automatically (center-point detection) using EthoVision XT 11.5 software (Noldus Information Technology, Leesburg, VA). The percentage of open arm entries (open arm entries/total arm entries) and percentage time on the open arms (open arm time/total time on the arms) were calculated. Heatmaps were produced with the EthoVision heatmap generator. The apparatus was cleaned with a Nolvasan solution (chlorhexidine diacetate) between tests.

After the elevated plus-maze test, locomotor activity was measured in the small open field for 10 min. The small open-field test (SOF) was conducted as described before (Qi et al., 2016; Bruijnzeel et al., 2022). The small open field test was conducted to assess locomotor activity, rearing, and stereotypies. These motor behaviors were measured using an automated animal activity cage system (VersaMax Animal Activity Monitoring System, AccuScan Instruments, Columbus, OH, United States). Horizontal beam breaks and total distance traveled reflect locomotor activity and vertical beam breaks reflect rearing. The distance traveled is dependent on the path of the animal in the open field and is considered a better indicator of locomotor activity than horizontal beam breaks. Repeated interruptions of the same beam are a measure of stereotypies (stereotypy count) (Calma et al., 2021). The setup consisted of four animal activity cages made of clear acrylic (40 cm × 40 cm × 30 cm; L × W × H), with 16 equally spaced (2.5 cm) infrared beams across the length and width of the cage. The beams were located 2 cm above the cage floor (horizontal activity beams). An additional set of 16 infrared beams were located 14 cm above the cage floor (vertical activity beams). All beams were connected to a VersaMax analyzer, which sent information to a computer that displayed beam data through Windows-based software (VersaDat software). The small open field test was conducted in a dark room, and the cages were cleaned with a Nolvasan solution between animals. At the beginning of each test, the rats were placed in the center of the small open field, and activity was measured.

#### 2.8.2 Withdrawal day 2: large open field test

The large open field test was conducted 48 h after the last nicotine and saline self-administration session. The large open field test is used to assess locomotor activity and anxiety-like behavior. The test was conducted for 10 min in a dimly lit room (75 lux), as described previously (Knight et al., 2021). The large open field apparatus consisted of a large arena measuring 120 × 120 × 60 cm (L × W × H). The arena was made of black high-density polyethylene panels that were fastened together and placed on a plastic bottom plate (Faulkner Plastics, Miami, FL). The rats’ behavior was recorded with a camera mounted above the arena and analyzed with EthoVision XT 11.5 software (Noldus Information Technology, Leesburg, VA). The large open field was divided into three zones: an outside zone (20 cm wide), a middle zone (20 cm wide), and a center zone (40 × 40 cm; L × W). The following behaviors were analyzed: total distance traveled, distance traveled in each zone (outside, middle, and center), number of entries into each zone, and latency to enter the middle and center zone. Rats avoid open spaces and therefore spend most of their time in the outside zone. Rats spend more time in the middle zone compared to the center zone, suggesting that the middle zone is less aversive than the center zone (Hernandez et al., 2021; Knight et al., 2021). Large open field heatmaps were produced with the EthoVision heatmap generator. The large open field was cleaned between rats with a Nolvasan solution.

#### 2.8.3 Withdrawal days 3–8: sucrose preference test

During the first withdrawal day, the rats were habituated to a pair of 180 mL Kaytee Chew Proof water bottles. The bottles were placed on top of the home cage and served as the only fluid source. During the second withdrawal day, the rats were habituated to two bottles containing a 2% (w/v) sucrose solution. These bottles were also placed on top of the home cage, and they were the sole source of fluid. Sucrose preference was measured for 5 days during nicotine withdrawal. The test was done using a two-bottle choice procedure, as described in our previous work (Bruijnzeel et al., 2019; Chellian et al., 2020). The sucrose solution (2%, w/v) was prepared daily with autoclaved water. One bottle with water and one bottle with a sucrose solution (2%, w/v) were placed on top of the home cage. The bottles were switched (left/right position) daily to reduce the side bias. The weight of each bottle was recorded before and after the 24-h choice test. The difference in bottle weights was used to measure water and sucrose intake. Sucrose preference (percentage) was calculated using the following formula: (sucrose intake/total fluid intake) × 100. After the sucrose preference test, the bottles were removed and water was available in the home cage.

### 2.9 Forced abstinence and nicotine intake

During the 2 weeks of forced abstinence, the rats were housed in the vivarium and were handled twice a week. After the 2-week forced abstinence period, the rats were allowed to self-administer nicotine (0.06 mg/kg/inf) and saline daily for 6 days under an FR1-TO60s schedule in 6-h sessions. During the 6-h self-administration sessions, the rats had access to water in the operant chambers.

### 2.10 Mecamylamine treatment, nicotine treatment, and nicotine intake

After six nicotine and saline self-administration sessions, the effects of mecamylamine on saline and nicotine self-administration (0.06 mg/kg/inf; FR1-TO60s schedule, 6 h) were investigated. Mecamylamine (0, 2 mg/kg, SC) was administered according to a Latin square design 10 min before the self-administration sessions. Seventy-two h after the last mecamylamine treatment, the effects of nicotine treatment on saline and nicotine self-administration (0.06 mg/kg/inf; FR1-TO60s schedule, 6 h) in rats was investigated. Nicotine (0, 0.4 mg/kg, SC) was administered according to a Latin square design 10 min before the self-administration sessions. Twenty-four and 48 h after each mecamylamine or nicotine treatment, saline and nicotine self-administration sessions were conducted without any drug treatment. Mecamylamine and nicotine treatment doses were based on one of our previous studies, which showed an effect on nicotine self-administration in rats 10 min after treatment but not at 24 and 48 h after treatment (Chellian et al., 2024). During the 6-h self-administration sessions, the rats had access to water in the operant chambers.

### 2.11 Forced abstinence and seeking behavior

During the 3 weeks of forced abstinence, the rats were housed in the vivarium and were handled twice a week. During the seeking tests, the rats were placed in the same operant chambers where they previously self-administered nicotine and saline. The operant sessions were conducted for 6 h under an FR1-TO60s schedule, without attaching the tethers to the vascular access buttons. Responding on the active lever turned on the pump for a 6.5-s period (saline or nicotine was not infused) and a cue light above the right lever, which remained illuminated throughout the time-out period. Responding on the inactive lever was recorded but did not have scheduled consequences. The active and inactive levers were retracted during the time-out period. Both active lever responses and effective active lever responses served as measures of seeking behavior. Active lever presses include both effective active lever presses, which turn on the pump and cue light, and ineffective active lever presses, which have no scheduled consequences (Kosten et al., 2004; Flagel et al., 2010). The ineffective active lever presses occur immediately after an active lever press and before the lever has been fully retracted. Following an effective active lever press, rats are exposed to the same cues (pump noise and visual cues) as they would be during a regular self-administration session but no nicotine or saline is infused. During the 6-h sessions, the rats had access to water in the operant chambers.

### 2.12 Statistics

Baseline (5 sessions) and long access (36 sessions) nicotine and saline self-administration data were analyzed using two- or three-way ANOVAs with hours, days, and session as within-subjects factors, and IVSA group and sex as between-subjects factors. Somatic withdrawal scores were analyzed with two- or three-way ANOVAs, with mecamylamine treatment and days as within-subjects factors, and IVSA group and sex as between-subjects factors. The effects of sex and nicotine self-administration on behavior in the elevated plus maze test, small open field, and large open field were analyzed using two- or three-way ANOVAs with IVSA group and sex as between-subjects factors. The effects of nicotine abstinence on sucrose preference were analyzed using three-way ANOVAs with days as a within-subjects factor, and IVSA group and sex as between-subjects factors. The effects of forced abstinence on nicotine intake and seeking were investigated using two- or three-way ANOVAs, with IVSA group and sex as between-subjects factors, and hours, days, and session as within-subjects factors. The effects of mecamylamine and nicotine treatment on nicotine intake were investigated using two- or three-way ANOVAs, with IVSA group and sex as between-subjects factors, and mecamylamine treatment, nicotine treatment, and hours as within-subjects factors. For all statistical analyses, significant interaction effects found in the ANOVAs were followed by Bonferroni’s *post hoc* tests to determine which groups differed. *p*-values less than or equal to 0.05 were considered significant. Data were analyzed with SPSS Statistics version 29 and GraphPad Prism version 10.1.2. The figures were generated using GraphPad Prism version 10.1.2. Large open field and elevated plus maze heatmaps were produced with the EthoVision heatmap generator.

## 3 Results

### 3.1 Baseline and thirty-six daily nicotine and saline self-administration sessions

#### 3.1.1 Week 5: baseline nicotine and saline self-administration

During the 1-h baseline self-administration sessions, infusions and active lever responses were higher for the rats that self-administered saline than for those that self-administered nicotine. Nicotine intake was the same in the males and the females during the baseline sessions (see Supplementary File S1 for results; Supplementary Figures S1A–H).

#### 3.1.2 Week 6–11: long access nicotine and saline self-administration

**FIGURE 2:**
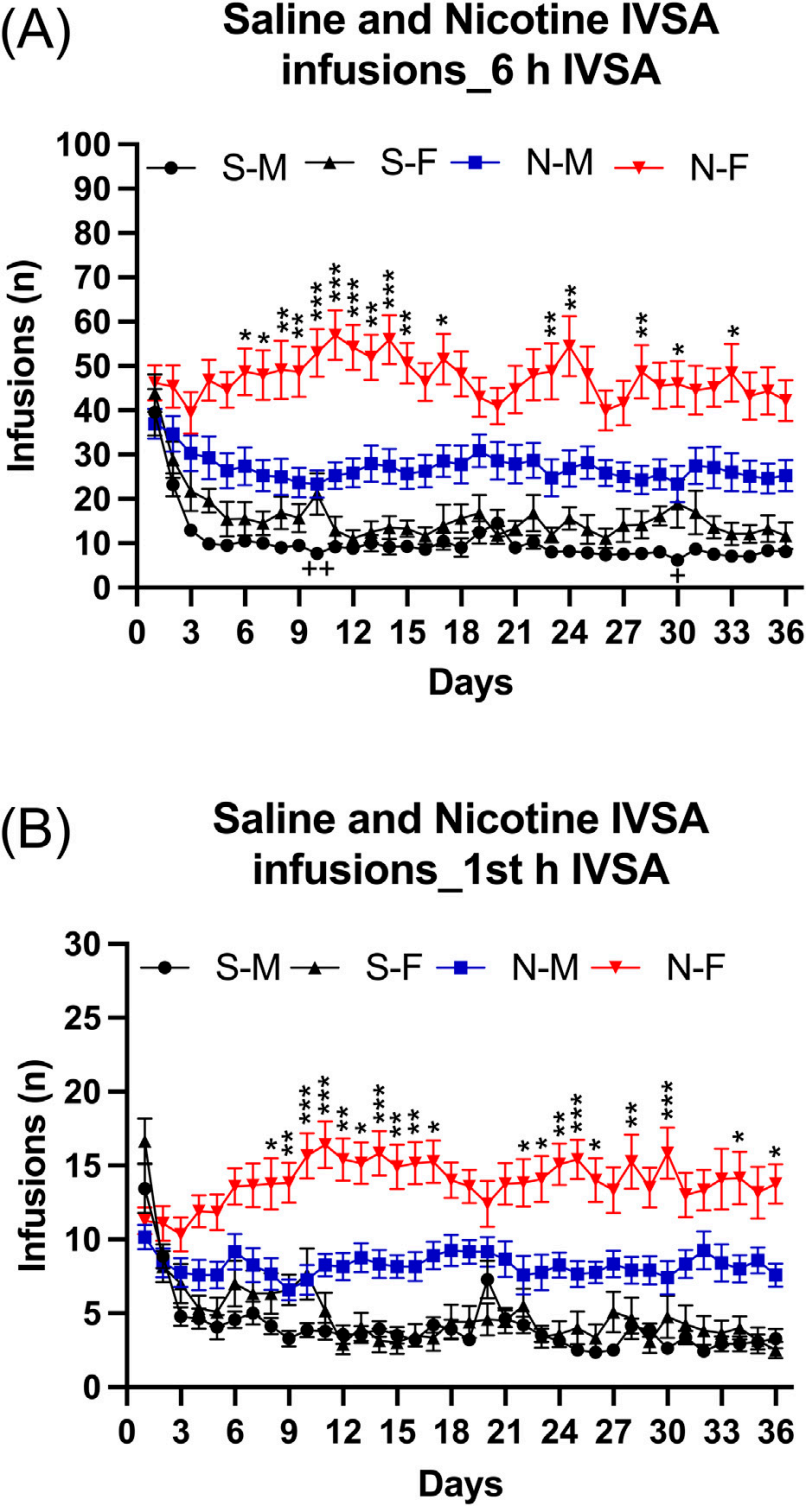
Daily 6-h nicotine and saline self-administration in male and female rats. The rats self-administered nicotine (0.06 mg/kg/inf) and saline in 6-h self-administration sessions for 36 days. Infusions **(A)** in 6-h nicotine and saline self-administration sessions. First hour infusions **(B)** in nicotine and saline self-administration groups. Asterisks indicate more infusions (nicotine intake) in the females that self-administered nicotine than in the males that self-administered nicotine on the same test day. Plus signs indicate more infusions in the females that self-administered saline than in the males that self-administered saline on the same test day. *, + *p* < 0.05; **, ++ *p* < 0.01; ***p < 0.001. Abbreviations and group size: S-M, saline IVSA-male (N = 14); N-M, nicotine IVSA-male (N = 12); S-F, saline IVSA-female (N = 12); N-F, nicotine IVSA-female (N = 12). Data are expressed as means ± SEM.

Infusions: Infusions were higher for the rats that self-administered nicotine than for those that self-administered saline (Figure 2A: IVSA group F1,46 = 62.478, *p* < 0.001). Infusions were much higher for the females that self-administered nicotine than for the males that self-administered nicotine and there was also a small sex difference in the saline groups (Figure 2A: Sex F1,46 = 18.03, *p* < 0.001; Sex × IVSA group F1,46 = 5.86, *p* < 0.05). The *post hoc* tests revealed that nicotine infusions (intake) in the female nicotine group were higher from day 6 compared to the male nicotine group (Figure 2A). However, on only 2 days (day 10 and 30) were infusions higher in the female saline group than in the male saline group (Figure 2A). In addition, the *post hoc* tests showed that the males and females in the nicotine group had more infusions than the males and females in the saline group (Supplementary Table S1). Infusions were stable in the females that self-administered nicotine but decreased in all other groups (Figure 2A; Supplementary Table S2: Days F35, 1610 = 11.547, *p* < 0.001; Days × Sex F35,1610 = 2.624, *p* < 0.001; Days × IVSA group F35,1610 = 6.602, *p* < 0.001; Days × Sex × IVSA group F35, 1610 = 2.128, *p* < 0.001).

Active lever: Active lever responses were higher for the rats that self-administered nicotine than for those that self-administered saline (see Supplementary File S2 for results; Supplementary Figure S2A; Supplementary Table S1, S2).

Inactive lever: Inactive lever responses declined more in the rats that self-administered saline than in the rats that self-administered nicotine and stabilized at a lower level in the saline rats than in the nicotine rats (see Supplementary File S2 for results; Supplementary Figure S2B; Supplementary Table S1, S2).

##### 3.1.2.1 Long access first hour infusions

During the first hour of access, rats had more nicotine than saline infusions (Figure 2B, IVSA group F1,46 = 68.433, *p* < 0.01). Furthermore, the female rats had more nicotine infusions compared to the males, but there was no sex difference in saline infusions (Sex F1,46 = 16.967, *p* < 0.001; Sex × IVSA group F1,46 = 9.447, *p* < 0.05). The *post hoc* tests showed that nicotine infusions (intake) in the female group were significantly higher from day 8 than in the males (Figure 2B). In addition, the *post hoc* tests showed that the males and females in the nicotine group had more infusions than males and females in the saline group (Supplementary Table S1). The number of infusions decreased over time in rats with access to saline, but remained stable in rats with access to nicotine (Supplementary Table S2, Days F35,1610 = 7.925, *p* < 0.001; Days × Sex F35,1610 = 2.0, *p* < 0.001; Days × IVSA group F35,1610 = 11.88, *p* < 0.001). In all groups, there was a decrease in infusions except for the nicotine females in which the number of infusions increased (Supplementary Table S2, Days × Sex × IVSA group F35,1610 = 2.226, *p* < 0.001).

##### 3.1.2.2 Long access first and last session

**FIGURE 3:**
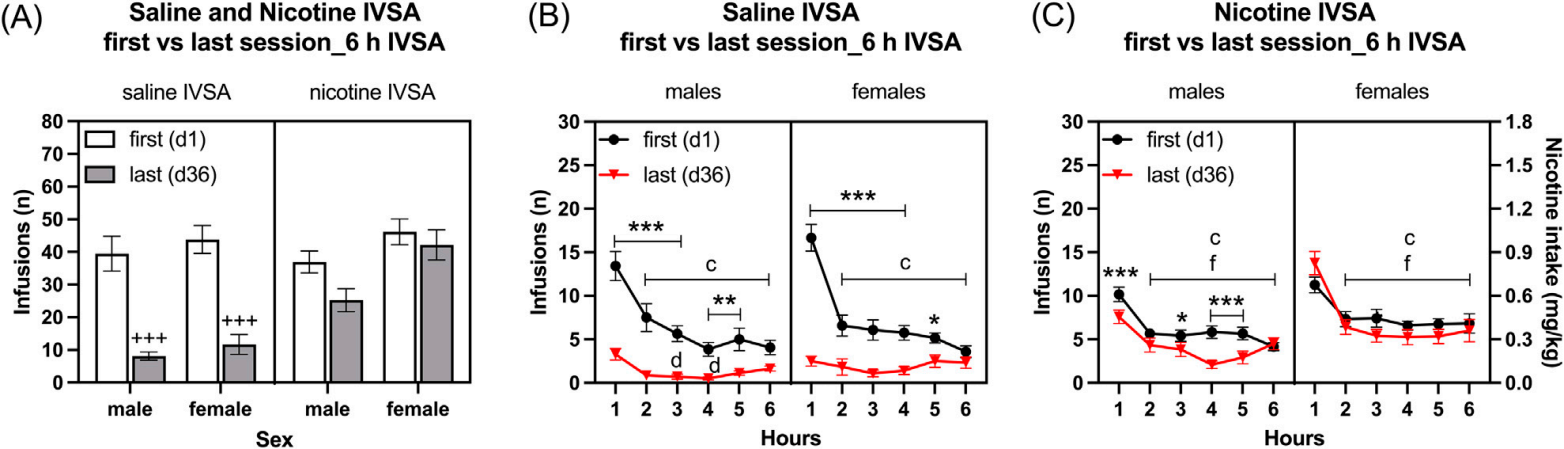
Comparison of the first and last nicotine and saline self-administration session in male and female rats. The rats self-administered saline and nicotine 0.06 mg/kg/inf in 6-h self-administration sessions for 36 days and compared their first (day 1) and last (day 36) self-administration sessions. Infusions **(A)** in 6-h nicotine and saline self-administration sessions. Time course of saline infusions **(B)** and nicotine infusions **(C)** during 6-h nicotine and saline self-administration sessions. Plus signs indicate a significant decrease in infusions in the last session compared to the first session with the same self-administration group and same sex. Asterisks indicate a significant difference in infusions between the first and last session in rats of the same sex, and at the same time point. Letter c indicates fewer infusions compared with first hour infusions in the first session with the same sex. Letters d and f indicate lower infusions compared with first hour infusions in the last session with the same sex. *, d *p* < 0.05; **p < 0.01; +++, ***, c, f *p* < 0.001. Saline IVSA-male (N = 14), nicotine IVSA-male (N = 12), saline IVSA-female (N = 12), and nicotine IVSA-female (N = 12). Data are expressed as means ± SEM.

Infusions: Rats that self-administered nicotine had more infusions responses compared to those that self-administered saline (Figure 3A: IVSA group F1,46 = 13.643, *p* < 0.001). Additionally, female rats had more infusions than male rats (Figure 3 As: Sex F1,46 = 7.042, *p* < 0.05; Sex × IVSA group F1, 46 = 2.039, NS). The number of infusions decreased from the first to the last session, with the greatest decrease in the saline group (Figure 3A: Session F1, 46 = 84.927, *p* < 0.001; Session × Sex F1, 46 = 0.637, NS; Session × IVSA group F1,46 = 31.016, *p* < 0.001; Session × Sex × IVSA group F1, 46 = 0.973, NS). The *post hoc* showed that both the male and the female rats in the saline group had fewer infusions during the last session compared to the first session (Figure 3A).

Active lever: Rats that self-administered nicotine had more infusions and active lever responses compared to those that self-administered saline (see Supplementary File S3 for results; Supplementary Figure S3A).

Inactive lever: Responding on the inactive lever decreased more from the first to the last session in the saline rats than in the nicotine rats (see Supplementary File S3 for results; Supplementary Figure S3B).

##### 3.1.2.3 Time course analysis, first day compared to last self-administration day

Saline self-administration group: Saline infusions were higher during the first session than the last session and they decreased over time (1–6 h) (Figure 3B, Session F1, 24 = 92.888, *p* < 0.01; Hours F5,120 = 45.335, *p* < 0.001). There were no effects of sex on saline infusions (Sex F1, 24 = 0.842, NS; Session × Sex F1, 24 = 0.015, NS; Hours × Sex F5, 120 = 0.688, NS). Furthermore, saline infusions were lower in both males and females during the last session compared to the first session (Session × Hours F5, 120 = 27.475, *p* < 0.001; Session × Hours × Sex F5, 120 = 2.457, *p* < 0.05). The *post hoc* analysis showed that, compared to the same time points during the first session, saline infusions in males and females were lower from the first hour to the fifth hour of the last session (Figure 3B). In addition, the *post hoc* analysis revealed that for both males and females, saline infusions were lower from the second to the sixth hour compared to the first hour during the first session. However, the *post hoc* showed that only for males the saline infusions were lower during the third and fourth hour compared to the first hour during the last sessions (Figure 3B).

Nicotine self-administration group: Nicotine infusions (intake) were higher during the first than the last session and it decreased over time (1–6 h) (Figure 3C, Session F1, 22 = 8.458, *p* < 0.01; Hours F5, 110 = 64.7, *p* < 0.001). Females self-administered more nicotine than the males (Sex F1, 22 = 7.434, *p* < 0.05; Session × Sex F1, 22 = 2.025, NS; Hours × Sex F5, 110 = 1.737, NS). Furthermore, nicotine infusions were lower in males during the last session compared to the first session, but in the females, it remained the same during the first and last sessions (Session × Hours F5, 110 = 3.22, *p* < 0.01; Session × Hours × Sex F5, 110 = 3.994, *p* < 0.01). The *post hoc* analysis showed that, compared to the same time points during the first session, nicotine infusions in males were lower during the first hour and the third to fifth hour of the last session (Figure 3C). In addition, the *post hoc* showed that for both males and females nicotine infusions were lower from the second to the sixth hour compared to the first hour during both the first and last sessions (Figure 3C).

### 3.2 Week 6–11: mecamylamine-precipitated somatic withdrawal signs

**FIGURE 4:**
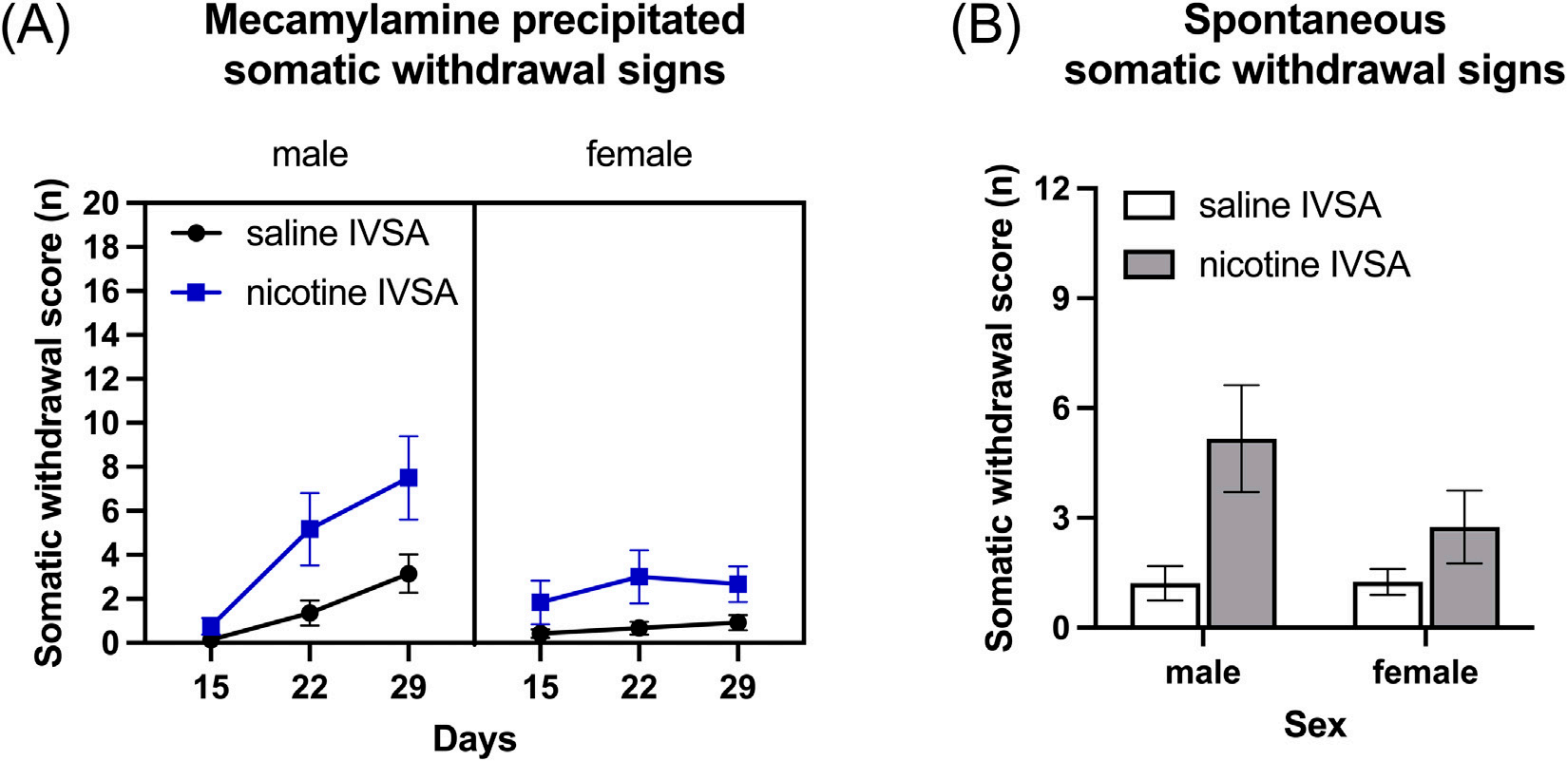
Mecamylamine-precipitated somatic withdrawal signs and spontaneous somatic withdrawal signs in male and female rats. Somatic withdrawal signs were recorded after mecamylamine administration **(A)** and after cessation of nicotine intake **(B)**. Saline IVSA-male (N = 14), nicotine IVSA-male (N = 12), saline IVSA-female (N = 12), and nicotine IVSA-female (N = 12). Data are expressed as means ± SEM.

Treatment with mecamylamine induced more somatic withdrawal signs in the rats that self-administered nicotine than in those that self-administered saline (Figure 4A, IVSA group F1, 46 = 9.086, *p* < 0.01). Furthermore, the number of somatic withdrawal signs increased over time and this effect was greater in the nicotine group than in the saline group (Figure 4A, Days F2, 92 = 97.894, *p* < 0.001; Sex F1, 46 = 3.267, NS; Days × Sex F2, 92 = 11.936, *p* < 0.001; Days × IVSA group F2, 92 = 3.779, *p* < 0.05; Sex × IVSA group F1, 46 = 0.478, NS; Days × Sex × IVSA group F2, 92 = 2.042, NS).

### 3.3 Week 11: spontaneous nicotine withdrawal

#### 3.3.1 Withdrawal day 1: spontaneous somatic withdrawal signs

Somatic withdrawal signs were assessed 16–17 h after the last self-administration session. The rats that self-administered nicotine displayed more somatic withdrawal signs than the rats that self-administered saline and there was no effect of sex (Figure 4B, Sex F1, 46 = 1.732, NS; IVSA group F1, 46 = 9.081, *p* < 0.01; Sex × IVSA group F1, 46 = 1.837, NS).

#### 3.3.2 Withdrawal day 1: elevated plus maze test

Nicotine abstinence did not increase anxiety-like behavior in either males or females (see Supplementary File S4 for results; Supplementary Figure S4). In comparison to the males, the females displayed less anxiety-like behavior in the elevated plus maze test (see Supplementary File S4 for results; Supplementary Figure S4). Heatmaps for both males and females in the nicotine and saline groups from the elevated plus-maze test are presented in Supplementary Figure S5.

#### 3.3.3 Withdrawal day 1: small open field test

**FIGURE 5:**
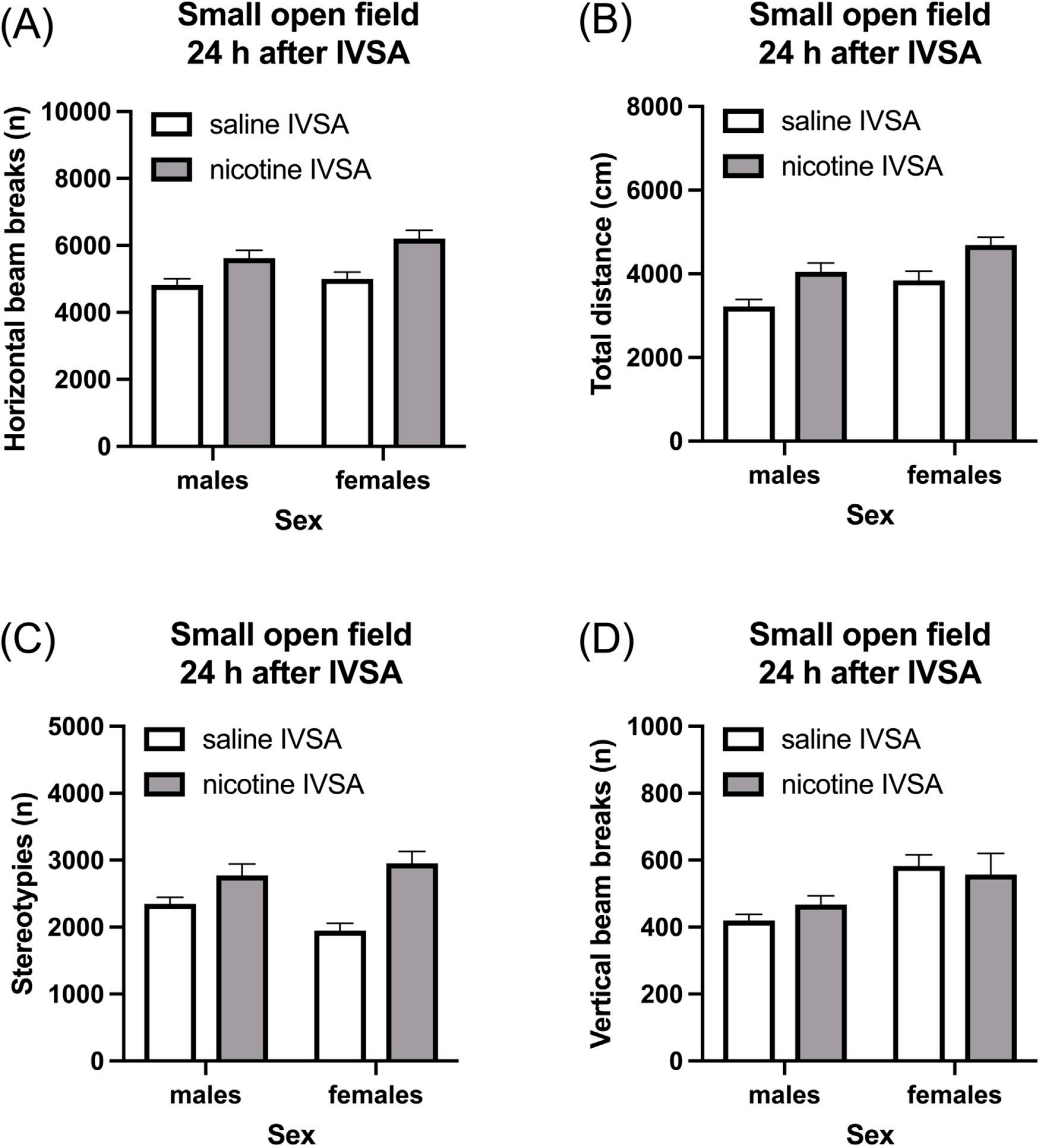
Nicotine withdrawal and locomotor activity in male and female rats. Nicotine withdrawal increases locomotor activity **(A, B)** and stereotypies **(C)** in the small open field test. Nicotine withdrawal does not affect the vertical beam breaks in the small open field test **(D)**. Saline IVSA-male (N = 14), nicotine IVSA-male (N = 12), saline IVSA-female (N = 12), and nicotine IVSA-female (N = 12). Data are expressed as means ± SEM.

Nicotine abstinence led to an increase in horizontal beam breaks (Figure 5A, IVSA group F1, 46 = 21.095, *p* < 0.001; Sex × IVSA group F1,46 = 0.817, NS), total distance traveled (Figure 5B, IVSA group F1,46 = 18.387, *p* < 0.001; Sex × IVSA group F1, 46 = 0.001, NS), and stereotypies (Figure 5C, IVSA group F1, 46 = 25.431, *p* < 0.001; Sex × IVSA group F1, 46 = 4.229, NS) and this was not affected by the sex of the rats. Nicotine abstinence did not affect vertical beam breaks (Figure 5D, IVSA group F1, 46 = 0.081, NS; Sex × IVSA group F1, 46 = 0.919, NS). Compared to the males, the females traveled a greater distance (Figure 5B, Sex F1, 46 = 10.495, *p* < 0.01) and had more vertical beam breaks (Figure 5D, Sex F1, 46 = 11.018, *p* < 0.01). There was no sex difference in horizontal beam breaks (Figure 5A, Sex F1, 46 = 3.059, NS) and stereotypies (Figure 5C, Sex F1, 46 = 0.597, NS).

#### 3.3.4 Withdrawal day 2: large open field test

Nicotine withdrawal did not increase anxiety-like behavior in either males or females (see Supplementary File S5 for results; Supplementary Figure S6). The females displayed less anxiety-like behavior than the males in the large open field test (see Supplementary File S5 for results; Supplementary Figure S6). Heatmaps for both males and females in the nicotine and saline groups from the large open field test are presented in Supplementary Figure S7.

#### 3.3.5 Withdrawal days 3–8: sucrose preference test

Nicotine abstinence did not affect sucrose preference in either males or females (see Supplementary File S6 for results; Supplementary Figure S8).

### 3.4 Week 13: forced abstinence and nicotine intake

#### 3.4.1 Last self-administration day compared to first one after forced abstinence

**FIGURE 6:**
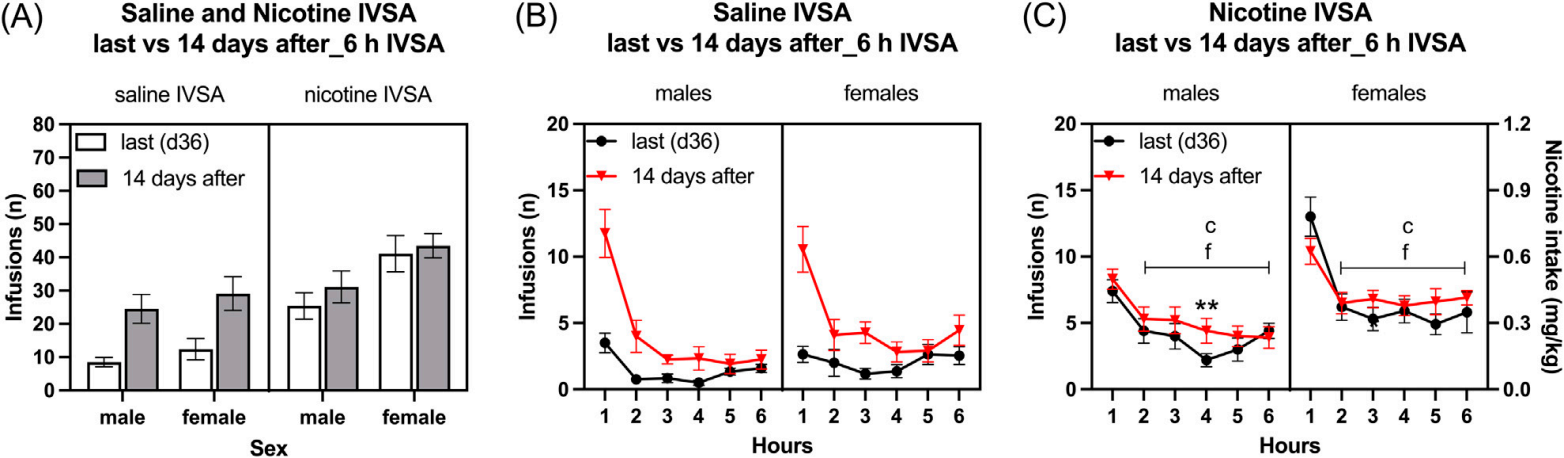
Nicotine and saline self-administration immediately after forced abstinence in male and female rats. Fourteen days after last self-administration (day 36), the rats self-administered saline and nicotine 0.06 mg/kg/inf in 6-h self-administration sessions. Infusions **(A)** in 6-h nicotine and saline self-administration sessions. Time course of saline infusions **(B)** and nicotine infusions **(C)** during 6-h nicotine and saline self-administration sessions. Asterisks indicate more infusions after the abstinence period in rats in the same sex and at the same time point. Letter c indicates fewer infusions compared with first-hour infusions in the last session within the same sex. Letters f indicates fewer infusions compared with first-hour infusions after the abstinence period (14 days after session) within the same sex. **p < 0.01; c, f *p* < 0.001. Saline IVSA-male (N = 12); nicotine IVSA-male (N = 10); saline IVSA-female (N = 11); nicotine IVSA-female (N = 10). Data are expressed as means ± SEM.

Infusions: The period of abstinence led to an increase in the number of infusions and this increase was greater in the rats that self-administered saline than in those that self-administered nicotine (Figure 6A: Session F1, 39 = 28.832, *p* < 0.001; IVSA group F1, 39 = 20.783, *p* < 0.001; Session × IVSA group F1,39 = 10.491, *p* < 0.01). The female rats had more infusions than the males (Figure 6A: Sex F1, 39 = 6.252, *p* < 0.05). The effect of sex on infusions was unaffected by the abstinence period, as well as by whether nicotine or saline was being self-administered (Figure 6A: Session × Sex F1, 39 = 0.114, NS; Sex × IVSA group F1, 39 = 1.806, NS; Session × Sex × IVSA group F1, 39 = 0.281, NS).

Active lever: The period of abstinence led to an increase in the number of active lever presses and this increase was greater in the rats that self-administered saline than in those that self-administered nicotine (see Supplementary File S7.1 for results; Supplementary Figure S9A)

Inactive lever: The rats that self-administered nicotine had more inactive lever responses than the rats that self-administered saline (see Supplementary File S7.1 for results; Supplementary Figure S9B)

#### 3.4.2 Time course analysis, last self-administration day compared to first one after forced abstinence

Saline self-administration group: Saline infusions were higher after the abstinence period than the last session and decreased over time (1–6 h) (Figure 6B, Session F1, 21 = 34.887, *p* < 0.001; Hours F5,105 = 39.55, *p* < 0.001; Session × Hours F5, 105 = 17.166, *p* < 0.001). There was no sex difference in saline infusions before and after the abstinence period (Sex F1, 21 = 0.88, NS; Session × Sex F1, 21 = 0.017, NS; Hours × Sex F5, 105 = 2.097, NS; Session × Hours × Sex F5,105 = 0.684, NS).

Nicotine self-administration group: Nicotine infusions (intake) were higher in female rats compared to males (Figure 6C, Sex F1, 18 = 5.692, *p* < 0.05). There was no significant difference in nicotine intake before and after the abstinence period (Session F1, 18 = 2.54, NS; Session × Sex F1, 18 = 0.422, NS). Nicotine infusions decreased over time, and this decrease was dependent on the sex of the rats (Hours F5, 90 = 57.595, *p* < 0.001; Hours × Sex F5,90 = 3.057, *p* < 0.05; Session × Hours F5, 90 = 2.121, NS; Session × Hours × Sex F5, 90 = 2.413, *p* < 0.05). The *post hoc* showed that nicotine infusions decreased over time in all groups. Furthermore, the *post hoc* showed that the nicotine infusions were higher during the fourth hour in the nicotine group after the 14-day abstinence period than before the abstinence period (Figure 6C).

#### 3.4.3 Self-administration after forced abstinence, six self-administration sessions

**FIGURE 7:**
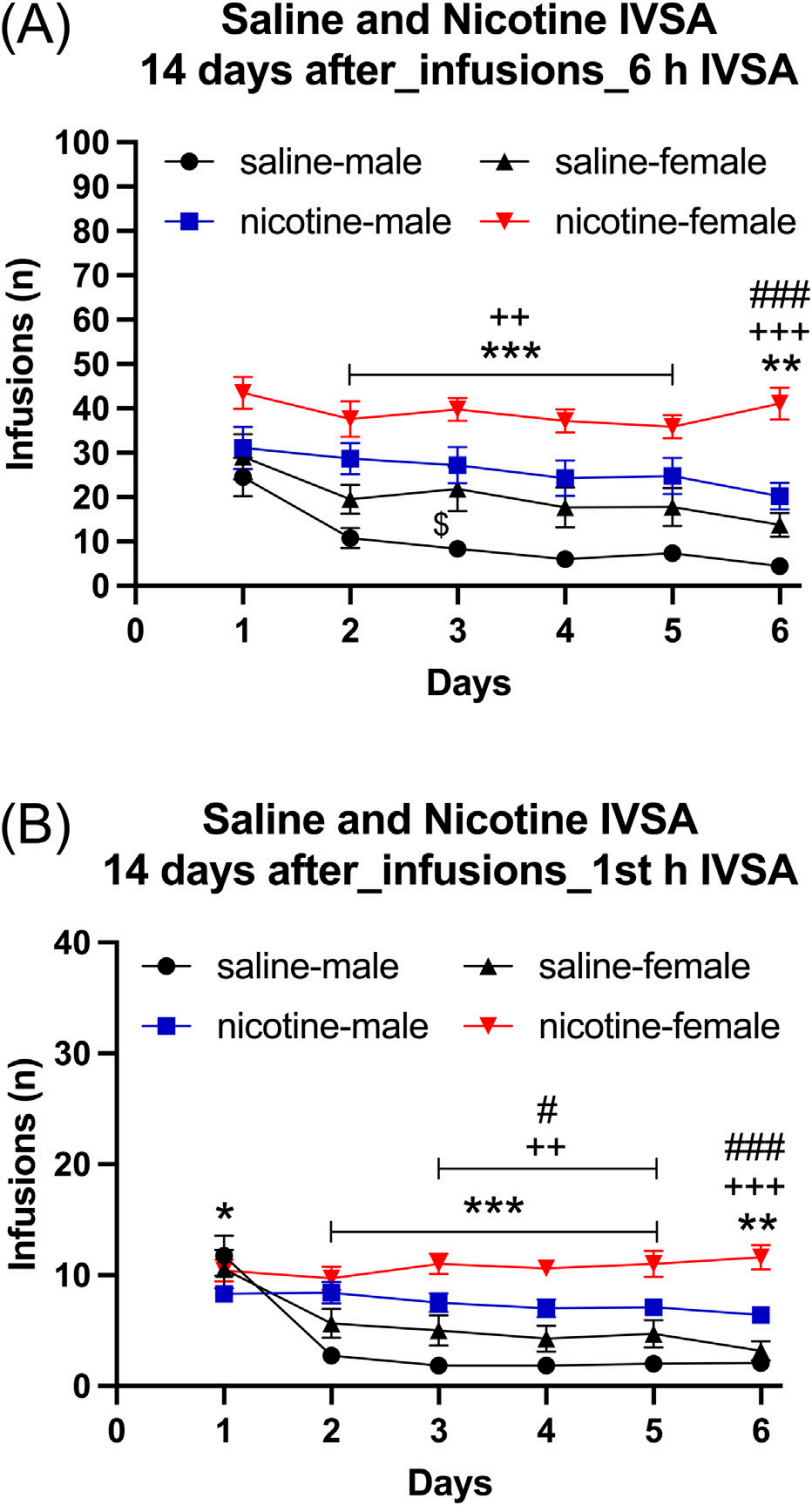
Daily nicotine and saline self-administration after forced abstinence in male and female rats. After a 14-day abstinence period, the rats self-administered saline and 0.06 mg/kg/inf of nicotine in 6-h self-administration sessions for 6 days. Infusions **(A)** in 6-h nicotine and saline self-administration sessions. First hour infusions **(B)** in nicotine and saline self-administration groups. Asterisks indicate more infusions in the nicotine group males than in the saline group males on the same day **(A, B)**. Plus signs indicate more infusions in the nicotine group females than in the saline group females at the same day **(A, B)**. Dollar signs indicate more infusions in the saline group females than in the saline group males on the same day **(A)**. Pound sign indicate more nicotine infusions in females than in males that self-administered nicotine on the same day **(A, B)**. *, $, #*p* < 0.05; **, ++ *p* < 0.01; ***, +++, ###*p* < 0.001. Saline IVSA-male (N = 12); nicotine IVSA-male (N = 10); saline IVSA-female (N = 11); nicotine IVSA-female (N = 10). Data are expressed as means ± SEM.

Infusions: The females had more infusions than the males (Figure 7A: Sex F1,39 = 13.859, *p* < 0.001). Furthermore, infusions were higher for the rats that self-administered nicotine than for those that self-administered saline (Figure 7A: IVSA group F1,39 = 32.433, *p* < 0.01; Sex × IVSA group F1,39 = 0.317, NS). Infusions decreased over time, and this effect was greatest in the males that self-administered saline (Figure 7A: Days F5, 195 = 26.077, *p* < 0.001; Days × Sex F5,195 = 2.259, *p* = 0.058; Days × IVSA group F5, 195 = 4.662, *p* < 0.001; Days × Sex × IVSA group F5, 195 = 2.182, *p* = 0.058). The *post hoc* tests showed that infusions were higher in the nicotine group than in the saline group (Figure 7A). Furthermore, females in both the nicotine and saline group had more infusions compared to the males (Figure 7A).

Active lever: The active lever responses were higher for the rats that self-administered nicotine than for those that self-administered saline (see Supplementary File S7.2 for results; Supplementary Figure S10A)

Inactive lever: The rats that self-administered saline had fewer inactive lever responses than the rats that self-administered nicotine (see Supplementary File S7.2 for results; Supplementary Figure S10B)

#### 3.4.4 Self-administration after forced abstinence, first hour infusions during six self-administration sessions

Female rats had more infusions than male rats (Figure 7B, Sex F1, 39 = 10.067, *p* < 0.01). Additionally, rats that self-administered nicotine had a higher number of infusions compared to those that self-administered saline (IVSA group F1, 39 = 30.416, *p* < 0.001; Sex × IVSA group F1, 39 = 0.769, NS). There was a decrease in the number of infusions over time, with the most noticeable reduction observed in male rats that self-administered saline (Days F5, 195 = 25.687, *p* < 0.001; Days × Sex F5, 195 = 2.895, *p* < 0.05; Days × IVSA group F5, 195 = 21.698, *p* < 0.001; Days × Sex × IVSA group F5, 90 = 2.383, *p* < 0.05). The *post hoc* test showed that the rats in the nicotine group had a higher number of infusions than the rats in the saline group (Figure 7B). Furthermore, the females had more nicotine, but not saline, infusions compared to the males (Figure 7B).

### 3.5 Week 14: mecamylamine treatment and self-administration

**FIGURE 8:**
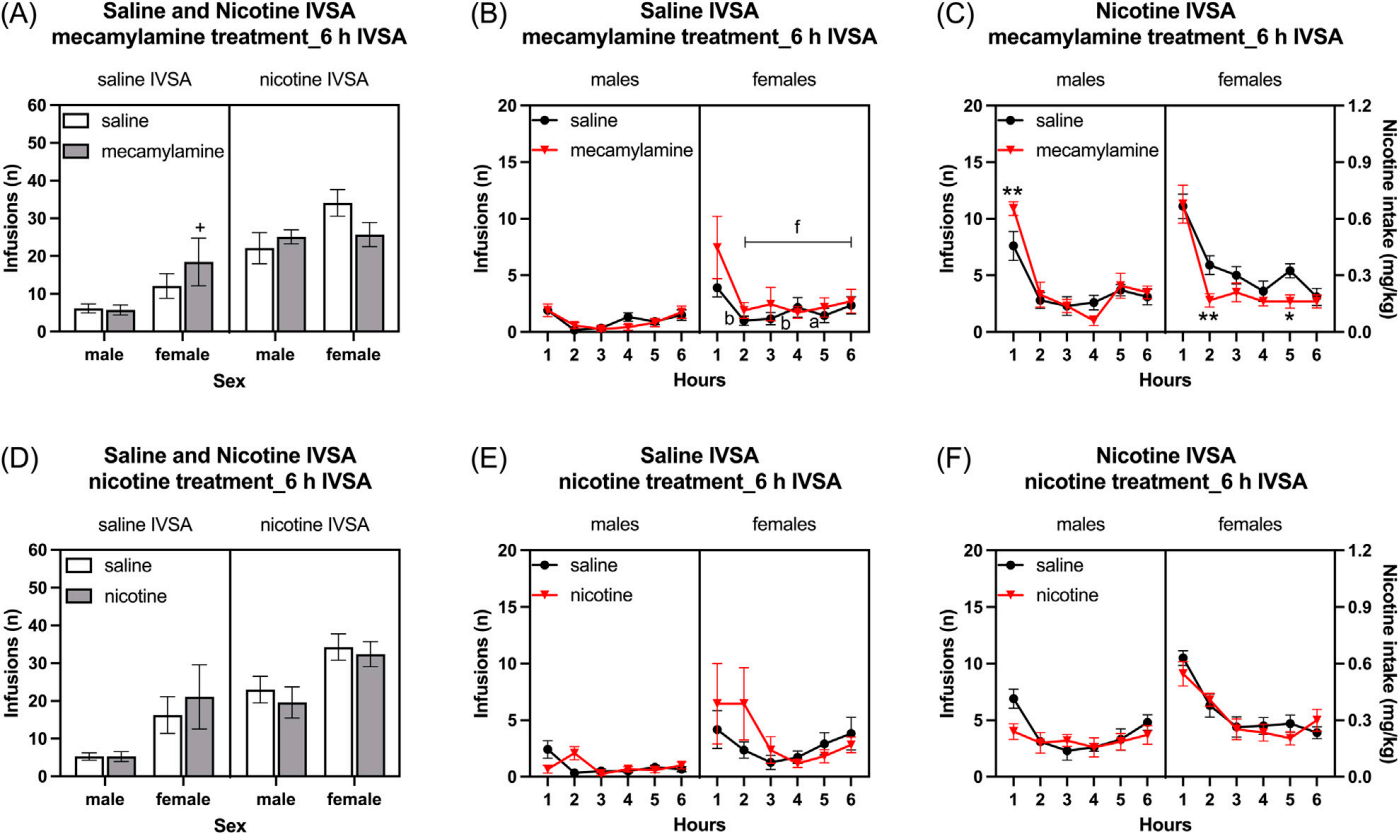
Effects of mecamylamine and nicotine treatment on nicotine and saline self-administration in male and female rats. Infusions **(A)** in 6-h nicotine and saline self-administration sessions after mecamylamine treatment. Time course of saline infusions **(B)** and nicotine infusions **(C)** during 6-h nicotine and saline self-administration sessions after mecamylamine treatment. Infusions **(D)** in 6-h nicotine and saline self-administration sessions after nicotine treatment. Time course of saline infusions **(E)** and nicotine infusions **(F)** during 6-h nicotine and saline self-administration sessions after nicotine treatment. The plus signs indicate more infusions in rats treated with mecamylamine compared to those treated with the vehicle, within the same self-administration group and sex. Asterisks indicate a difference in infusions between rats treated with mecamylamine and vehicle within the same sex, and time point. Letters a and b indicate lower infusions compared with first hour infusions in vehicle-treated rats with the same sex. Letter f indicates lower infusions compared with first hour infusions in mecamylamine-treated rats with the same sex. +, *, a *p* < 0.05; **, b *p* < 0.01; f *p* < 0.001. Saline IVSA-male (N = 12), nicotine IVSA-male (N = 10), saline IVSA-female (N = 11), and nicotine IVSA-female (N = 10). Data are expressed as means ± SEM.

Infusions: Infusions were higher in females than in males, and they were higher in rats that self-administered nicotine compared to those that self-administered saline (Figure 8A: Sex F1, 39 = 6.484, *p* < 0.05; IVSA group F1, 39 = 27.691, *p* < 0.001). Mecamylamine decreased the number of infusions in the females that self-administered nicotine. However, mecamylamine increased the number of infusions in the females that self-administered saline, while not affecting infusions in the males (Figure 8A: Mecamylamine treatment F1, 39 = 0.008, NS; Mecamylamine treatment × Sex F1, 39 = 0.546, NS; Mecamylamine treatment × IVSA group F1, 39 = 3.294, NS; Sex × IVSA group F1, 39 = 0.242, NS; Mecamylamine treatment × Sex × IVSA group F1, 39 = 8.455, *p* < 0.01). The *post hoc* analysis showed that infusions were higher in mecamylamine-treated females that self-administered saline than in saline-treated females that self-administered saline (Figure 8A).

Active lever: Mecamylamine decreased the active lever responses in the females that self-administered nicotine. However, it increased the active lever responses in the females that self-administered saline, while not affecting active lever responses in the males (see Supplementary File S8 for results; Supplementary Figure S11A).

Inactive lever: Mecamylamine treatment increased inactive lever responses and this parameter was not affected by the sex of the rats (see Supplementary File S8 for results; Supplementary Figure S11B).

#### 3.5.1 Time course analysis, mecamylamine treatment and self-administration

Saline self-administration group: Mecamylamine administration did not affect the saline infusions in both males and females (Figure 8B, Mecamylamine treatment F1, 21 = 2.994, NS; Mecamylamine treatment × Sex F1, 21 = 3.892, NS; Mecamylamine treatment × Hours F5, 105 = 2.102, NS; Mecamylamine treatment × Hours × Sex F5, 105 = 1.322, NS). There was no sex difference in saline infusions (Sex F1, 21 = 3.964, NS). However, saline infusions decreased over time only in females (Hours F5, 105 = 11.037, *p* < 0.001; Hours × Sex F5, 105 = 2.926, *p* < 0.05). The *post hoc* showed that in females, saline infusions were lower from the second hour in both the saline and mecamylamine treatment groups (Figure 8B).

Nicotine self-administration group: Treatment with mecamylamine differently affected nicotine infusions (intake) in females and males (Figure 8C, Mecamylamine treatment F1, 18 = 0.99, NS; Mecamylamine treatment × Sex F1, 18 = 4.413, *p* < 0.05). The *post hoc* analysis showed that treatment with mecamylamine increased nicotine infusions in the males and decreased nicotine intake in the females (Figure 8C). There was no sex difference in nicotine infusions (Sex F1, 18 = 2.789, NS). However, nicotine infusions decreased over time in both males and females (Hours F5, 90 = 62.916, *p* < 0.001; Hours × Sex F5, 90 = 1.787, NS). Moreover, the effect of mecamylamine on nicotine infusions was dependent on the time point (Mecamylamine treatment × Hours F5, 90 = 3.611, *p* < 0.01; Mecamylamine treatment × Hours × Sex F5, 90 = 1.796, NS).

### 3.6 Week 15: nicotine treatment and self-administration

Infusions: The administration of nicotine 10 minutes before the self-administration sessions did not affect the number of infusions (Figure 8D,: Nicotine treatment F1, 39 = 0.005, NS; Nicotine treatment × Sex F1, 39 = 0.777, NS; Nicotine treatment × IVSA group F1, 39 = 1.992, NS; Nicotine treatment × Sex × IVSA group F1, 39 = 0.214, NS). The female rats had a higher number of infusions compared to the males (Figure 8D: Sex F1, 39 = 10.476, *p* < 0.01). Furthermore, the rats that self-administered nicotine had more than the rats that self-administered saline and this was not affected by the sex of the rats (Figure 8D: IVSA group F1, 39 = 15.224, *p* < 0.001; Sex × IVSA group F1,39 = 0.031, NS).

Active lever: The administration of nicotine 10 minutes before the self-administration sessions did not affect the number of active lever responses (see Supplementary File S9 for results; Supplementary Figure S11C).

Inactive lever: Treatment with nicotine or the sex of the rats did not affect inactive lever responses (see Supplementary File S9 for results; Supplementary Figure S11D).

#### 3.6.1 Time course analysis, nicotine treatment and self-administration

Saline self-administration group: Saline infusions were higher in females than males (Figure 8E, Sex F1, 21 = 4.935, *p* < 0.05). Nicotine treatment affects the saline infusions in both males and females only at the beginning of the self-administration period (Figure 8E, Nicotine treatment F1, 21 = 0.678, NS; Nicotine treatment × Sex F1, 21 = 0.678, NS; Nicotine treatment × Hours F5, 105 = 3.105, *p* < 0.05; Nicotine treatment × Hours × Sex F5, 105 = 2.086, NS). Saline infusions decreased over time and there was no effect of sex (Hours F5, 105 = 3.618, *p* < 0.01; Hours × Sex F5, 105 = 1.193, NS).

Nicotine self-administration group: Nicotine infusions were higher in the females than the males (Figure 8F, Sex F1, 18 = 6.367, *p* < 0.05). Nicotine infusions decreased over time and this effect was greater in the females than the males (Hours F5, 90 = 39.32, *p* < 0.001; Hours × Sex F5, 90 = 9.761, <0.001). Treatment with nicotine slightly decreased nicotine infusions at the beginning of the session and increased infusions towards the end of the session (Figure 8F, Nicotine treatment F1, 18 = 2.06, NS; Nicotine treatment × Hours F5, 90 = 2.929, *p* < 0.05; Nicotine treatment × Sex F1, 18 = 0.165, NS; Nicotine treatment × Hours × Sex F5, 90 = 1.691, NS).

### 3.7 Week 18: forced abstinence and nicotine- and saline-seeking behavior

#### 3.7.1 Last self-administration day compared to seeking session after forced abstinence

**FIGURE 9:**
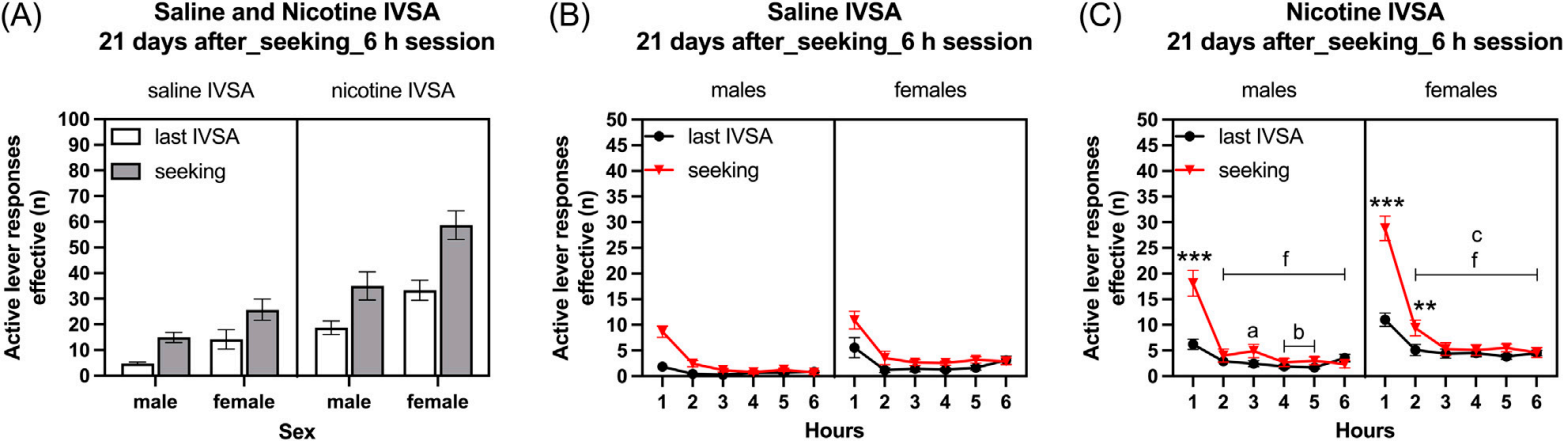
Nicotine and saline seeking behavior after forced abstinence in male and female rats. Twenty-one days after last self-administration session, the rats were placed in the operant chamber and the lever responses were recorded in a 6-h session. Effective active lever responses **(A)** in 6-h sessions. Time course of effective active lever responses in saline group **(B)** and nicotine group **(C)** rats during 6-h sessions. Asterisks indicate a significant difference in effective active lever responses between the seeking session and last self-administration session within the same sex and time point. The letters a, b, and c indicate fewer effective active lever responses compared with the first-hour time point in the last session within the same sex. The letter f indicates fewer effective active lever responses compared with first-hour effective active lever responses in the seeking session within the same sex. a *p* < 0.05; **, b *p* < 0.01; ***, c, f *p* < 0.001. Saline IVSA-male (N = 12); nicotine IVSA-male (N = 9); saline IVSA-female (N = 11); nicotine IVSA-female (N = 10). Data are expressed as means ± SEM.

Effective active lever: Rats that self-administered nicotine had more effective active lever responses than those self-administering saline (Figure 9A, IVSA group F1, 38 = 40.139, *p* < 0.001). After a period of abstinence, effective active lever presses increased more in rats with a history of nicotine self-administration compared to those with a history of saline self-administration (Session F1, 38 = 1112.318, *p* < 0.001; Session × IVSA group F1, 38 = 11.063, *p* < 0.001). Additionally, the females had more effective active lever presses than the males (Sex F1, 38 = 18.599, *p* < 0.001; Session × Sex F1, 38 = 2.988, NS; Sex × IVSA group F1,38 = 1.753, NS; Session × Sex × IVSA group F1,38 = 1.681, NS).

Active lever: Following a period of abstinence, active lever presses increased, and this effect was greater in the rats with a history of nicotine self-administration than in the rats with a history of saline self-administration (see Supplementary File S10 for results; Supplementary Figure S12A).

Inactive lever: The inactive lever presses increased after abstinence in the rats with a history of nicotine self-administration and saline self-administration (see Supplementary File S10 for results; Supplementary Figure S12B).

#### 3.7.2 Time course analysis, last self-administration day compared to seeking session after forced abstinence

Saline self-administration group: Effective active lever presses decreased over time and the females had more effective active lever presses than the males (Figure 9B, Hours F5, 105 = 48.894, *p* < 0.001; Sex F1, 21 = 7.087, *p* < 0.05). Effective active lever responses were higher after the abstinence period in rats of both sexes (Figure 9B, Session F1, 21, 58.602, *p* < 0.001; Session × Sex F1, 21, 0.207, NS). Over time, there was a decrease in effective active lever responses, and this decrease was more significant during the seeking session compared to the final self-administration session (Hours × Sex F5, 105 = 1.63, NS; Hours × Session F5, 105 = 13.088, *p* < 0.001; Hours × Session × Sex F5, 105 = 0.582, NS).

Nicotine self-administration group: Effective active lever presses decreased over time and the females had more effective active lever presses than the males (Figure 9C, Hours F5, 85 = 98.353, *p* < 0.001; Sex F1, 17 = 10.548, *p* < 0.01). Over time, the decrease in effective active lever responses was more pronounced in females than in males, and the decrease in responses was larger during the seeking session compared to the final self-administration session (Figure 9C, Hours × Sex F5, 85 = 5.82, *p* < 0.001; Hours × Session F5, 85 = 46.744, *p* < 0.001). Effective active lever responses were higher after the abstinence period in rats of both sexes (Session F1, 17, 54.649, *p* < 0.001; Session × Sex F1, 17, 2.579, NS). Despite the initial strong increase in effective active lever responses in females following the abstinence period, there was a rapid decline in effective active lever responses after the first hour of access to the operant chambers (Hours × Session × Sex F5, 85 = 2.592, *p* < 0.05). The *post hoc* showed that effective active lever responses were higher during the first hour in males and first and second hour in females compared to the last self-administration session. Additionally, effective active lever responses decreased over time in all groups (Figure 9C).

## 4 Discussion

These studies aimed to determine whether there are sex differences in nicotine intake, affective and somatic withdrawal signs, and relapse parameters in rats. Specifically, we investigated whether male and female rats with long access to nicotine or saline display differences in nicotine intake (infusions), somatic withdrawal signs, activity levels, anxiety-like behavior, anhedonia, and sensitivity to nicotine and mecamylamine. Additionally, we assessed whether there are sex differences in nicotine and saline intake, as well as in nicotine and saline intake after forced abstinence, and seeking behavior following a period of forced abstinence. Our findings revealed that female rats self-administer more nicotine than male rats. However, there was no sex difference in precipitated somatic withdrawal signs. In addition, following the cessation of nicotine self-administration, both male and female rats displayed spontaneous somatic withdrawal signs and were hyperactive in the small open field, with no significant sex differences observed. However, cessation of nicotine intake did not lead to an increase in anxiety-like behavior in the large open field test or in the elevated plus maze test. Additionally, cessation of nicotine intake did not cause anhedonia, as indicated by the absence of a difference in sucrose preference between the rats that self-administered nicotine or saline. A period of forced abstinence increased saline, but not nicotine, intake in the males and the females. Mecamylamine differently affected nicotine intake in the males and the females. In males, mecamylamine treatment increased nicotine intake, whereas in females, mecamylamine decreased nicotine intake. Operant responding for nicotine was not affected by a period of forced abstinence. However, nicotine seeking was increased after a period of forced abstinence. The females displayed more nicotine seeking than the males after the abstinence period. Taken together, these findings indicate that there are significant sex differences in nicotine intake, the effects of mecamylamine on nicotine intake, and nicotine seeking following a period of forced abstinence.

In the present study, we investigated sex differences in nicotine intake during both five 1-h and thirty-six 6-h self-administration sessions. During the initial five 1-h nicotine self-administration sessions (3 sessions with 0.03 mg/kg/inf of nicotine and 2 sessions with 0.06 mg/kg/inf of nicotine), there was no sex difference in nicotine intake. Contrary to the findings of this current study, in our previous work with adult rats, we found that nicotine intake was higher in females than males when the rats had access to 0.03 mg/kg/inf for 1 h per day (Chellian et al., 2021b; Chellian et al., 2021c). However, these studies differed from the present study in which the rats were trained to respond for food pellets before the nicotine self-administration sessions and then self-administered 0.03 mg/kg/inf of nicotine for 3 days. In one of the prior studies the sex differences in nicotine intake were only observed after the first 10 days of nicotine self-administration (Chellian et al., 2021b). In the other study, the rats did not receive any food training and the spontaneous acquisition of nicotine intake was investigated (Chellian et al., 2021c). Several other studies also reported no sex differences in nicotine intake when the rats have short access (1–2 h/day) to 0.03 mg/kg/inf of nicotine (Donny E. C. et al., 2000; Chaudhri et al., 2005; Feltenstein et al., 2012).

In the present study, there was no sex difference in nicotine intake during the two short access sessions where the rats had access to 0.06 mg/kg/inf of nicotine. However, nicotine intake was higher in the females than in the males when the rats had long access to 0.06 mg/kg/inf of nicotine. In line with the current study, one of our previous studies, in which the rats self-administered 0.06 mg/kg/inf of nicotine for 1 h per day, also did not detect a sex difference in nicotine intake (Chellian et al., 2023). Other studies reported that nicotine intake is higher in females than males with long access (23-h sessions) to 0.06 mg/kg/inf of nicotine (Grebenstein et al., 2013; Flores et al., 2016). A recent meta-analysis with 20 studies concluded that female rats self-administer more nicotine than male rats (Flores et al., 2019). This meta-analysis also showed that sex differences in nicotine self-administration are greater in animals with long than shorter access periods. This finding aligns with our observation that while no sex difference in nicotine intake was observed when the rats self-administered 0.06 mg/kg/inf of nicotine for 1 h a day, a robust significant sex difference was observed when the rats self-administered the same dose for 6 h per day (Chellian et al., 2023).

In the present study, the rats had a higher level of operant responding for nicotine than for saline. This corroborates previous studies that demonstrated that operant responding for nicotine is higher than for saline (Sanchez et al., 2014; Jin et al., 2020; Chellian et al., 2021b). These findings support the hypothesis that nicotine is a potent reinforcer in rodents (Stolerman and Jarvis, 1995; Balfour, 2008). In the present study, we also determined sex difference in the self-administration of saline. Interestingly, the females responded more for saline than the males. This higher level of responding for saline was observed during the first ten long access self-administration sessions and again during the second half of the 36-day self-administration period. This higher level of responding for saline in the females is not in line with prior studies that reported that there are no sex differences in the self-administration of saline (Sanchez et al., 2014; Johansen and McFadden, 2017). Several potential factors could explain the higher level of operant responding for saline in the female rats. Firstly, females are more active than males, which could potentially lead to more responding for saline (Knight et al., 2021). Secondly, prior to the saline self-administration sessions, the rats were trained to respond for food pellets and delivery of the food pellets was paired with a visual cue. Female rats are more responsive to cues associated with food rewards than males (Grimm et al., 2022). Therefore, it might be possible that the females responded more for saline because of their heightened responsiveness to cues paired with the delivery of food pellets.

In this study, we assessed both precipitated and spontaneous somatic withdrawal signs in male and female rats. Extensive evidence indicates that noncontingent exposure to nicotine via injections, minipumps, or tobacco smoke leads to the development of dependence, as indicated by spontaneous and precipitated somatic withdrawal signs (Epping-Jordan et al., 1998; Skjei and Markou, 2003; Small et al., 2010; Chellian et al., 2021a). The development of dependence has also been investigated in male rats that self-administer nicotine under long access conditions (Paterson and Markou, 2004; O'Dell et al., 2007; O'Dell and Koob, 2007; Cohen et al., 2012; Cohen et al., 2015). These studies indicate that both precipitated and spontaneous somatic withdrawal signs can be observed in rats with long access to nicotine (Chellian et al., 2022a). Our study expands on this work by examining precipitated and spontaneous withdrawal signs in both male and female rats and control rats that self-administer saline. Our findings show that rats that self-administer nicotine have more precipitated and spontaneous somatic withdrawal signs compared to those that self-administer saline, and there are no sex differences in precipitated and spontaneous somatic withdrawal signs. Interestingly, we also found that the number of precipitated somatic withdrawal signs in the nicotine group increased over time. This finding aligns with our previous study, which showed that mecamylamine-precipitated somatic withdrawal signs increase over time in rats exposed to tobacco smoke (Chellian et al., 2020). This pattern of results suggests that rats gradually develop nicotine dependence (Malin et al., 1992).

The rats were tested in the small open field test to determine if cessation of nicotine self-administration affects locomotor activity. Cessation of nicotine intake led to an increase in horizontal beam breaks, locomotor activity, and stereotypies in both males and females. The observation that cessation of nicotine self-administration increases locomotor activity aligns with the observation that removal of nicotine pumps leads to an increase in locomotor activity in male mice (Kota et al., 2007). However, the same group reported that removal of the nicotine pumps did not lead to an increase in locomotor activity in female mice (Kota et al., 2008). Numerous other studies reported that cessation of nicotine administration does not affect or decreases locomotor activity in rats and mice (Isola et al., 1999; Hamilton et al., 2009; Malin and Goyarzu, 2009). Interestingly, restlessness is a common symptom of smoking cessation in humans (Hughes and Hatsukami, 1986). Notably, in a recent study, restlessness (26.82%) was the second most commonly reported withdrawal symptom, following urges to smoke (34.01%) (Cui et al., 2023). The observed increase in locomotor activity in both male and female rats after cessation of nicotine intake could potentially serve as an animal model for studying the restlessness experienced during a smoking cessation attempt in humans.

To investigate whether cessation of nicotine self-administration leads to an increase in anxiety-like behavior, the rats were tested in the elevated plus maze test and in the large open field. Cessation of nicotine self-administration did not increase anxiety-like behavior in these tests. These outcomes were somewhat unexpected, as some previous studies reported that cessation of nicotine self-administration increases anxiety-like behavior. For example, cessation of long access (23 h) nicotine self-administration increases anxiety-like behavior in rats in the elevated plus maze test (Cohen et al., 2015). Similarly, cessation of noncontingent nicotine administration has been shown to increase anxiety-like behavior in the elevated plus maze test in both rats and mice (Pandey et al., 2001; Kotagale et al., 2015; Bagosi et al., 2016). However, it should be noted that other studies did not report an increase in anxiety-like behavior after the cessation of noncontingent nicotine administration or nicotine self-administration (Grabus et al., 2005; Biała et al., 2014; Zaniewska et al., 2021). Given these discrepancies in the effects of nicotine cessation on anxiety-like behavior, further research is needed to identify the specific factors that contribute to anxiety-like behavior following the cessation of nicotine administration.

In the present study, the females displayed less anxiety-like behavior than the males in the elevated plus maze, as indicated by a higher percentage of time on the open arms and a higher percentage of open arm entries. Furthermore, the females displayed less anxiety-like behavior in the large open field test, as indicated by a shorter latency to enter the center of the field and a longer duration spent in the center. These observations are in line with our previous work, in which we showed that females display less anxiety-like behavior than males in the large open field test and the elevated plus maze test (Knight et al., 2021).

In this study, we also investigated the effects of a period of forced abstinence on the self-administration of nicotine and saline. We found that the self-administration of saline, but not nicotine, increased after the period of forced abstinence. Several studies have investigated nicotine intake after a period of abstinence, but these studies did not include female rats (George et al., 2007; O'Dell and Koob, 2007). A study by George et al. (2007) demonstrated that rats, when given 23-h access to nicotine at a dose of 0.03 mg/kg/inf, have increased nicotine intake following a 3-day abstinence period (George et al., 2007). O'Dell and Koob (2007) also investigated the abstinence effect in rats (O'Dell and Koob, 2007). In their study, the rats were allowed to self-administer either saline or a gradually increasing dose of nicotine (0.015 mg/kg/inf to 0.09 mg/kg/inf). The rats had access to each nicotine dose for four 23-h sessions, with a 2-day break between each dose. Nicotine intake was highest during the initial self-administration session and subsequently declined. The rats stopped responding for saline after the first two self-administration sessions. There are some similarities and differences between the outcomes of the present study and those of previous studies that investigated the effects of forced abstinence on nicotine intake. In the present study, we found that nicotine intake was highest during the first session after the abstinence period and then gradually declined. Previous studies that investigated the effects of abstinence on nicotine intake reported a similar pattern with the highest nicotine intake in the first session after abstinence, followed by a gradual decline (George et al., 2007; O'Dell and Koob, 2007). However, we did not observe an increase in nicotine intake after the abstinence period as reported previously (George et al., 2007). There are several potential differences between the present study and the study by George et al. (2007) that might account for this discrepancy. Firstly, the nicotine dose was 0.06 mg/kg/inf in the present study and 0.03 mg/kg/inf in the study by George et al. (2007). Nicotine intake is higher when the rats have access to the 0.06 mg/kg/inf dose compared to when they have access to the lower 0.03 mg/kg/inf dose (Shoaib and Stolerman, 1999; Cohen et al., 2015). Other differences between the present study and the study by George et al. (2007) that might account for the differences in nicotine intake after abstinence include the duration of the self-administration sessions (23 h in their study *versus* 6 h in our study) and the duration of the abstinence period (3 days in their study *versus* 14 days in our study).

Previous work has shown that rats respond for saline when the delivery of saline is paired with a visual cue (Jin et al., 2020; Tapia et al., 2022; Stringfield et al., 2023). It was interesting to note that in the present study there was a robust increase in the intake of saline, but not nicotine, in the males and females after the forced abstinence period. This may suggest that the period of abstinence enhances the reinforcing properties of environmental cues, such as the visual cue paired with saline, which in turn increases responding for saline. It is also possible that the period of abstinence increased the reinforcing properties of the visual cue paired with nicotine, but this did not further increase nicotine intake because high doses of nicotine are aversive (Risinger and Oakes, 1995; Igari et al., 2013). It has been suggested that nicotine intake leads to satiety, and that after this satiety point has been reached, the motivation to self-administer nicotine decreases (Fowler and Kenny, 2014). Nicotine intake is rewarding up to the point of satiety, and intake beyond this point gradually becomes more aversive. It is possible that the period of forced abstinence does not change the satiety point and therefore abstinence does not lead to an increase in nicotine intake.

In this study, we also investigated the effects of 3 weeks of forced abstinence on nicotine and saline seeking. The abstinence period led to an increase in nicotine and saline seeking. A previous study suggested that nicotine and saline-seeking behavior is only observed in rats that received food-training prior to the self-administration sessions (Clemens et al., 2010). In addition, they found that food training results in similar levels of nicotine and saline seeking. This suggests that seeking behavior is primarily associated with food acting as a reinforcer (Clemens et al., 2010). However, in our study, the rats with a prior history of food training displayed significantly more nicotine-seeking than saline-seeking behavior. Therefore, our findings indicate that nicotine also acts as a reinforcer for seeking behavior. In our study, the females displayed more nicotine seeking than the males. This work builds upon prior research that investigated nicotine seeking after abstinence but did not include females or saline self-administration groups (Markou et al., 2018; Domi et al., 2023). We are unaware of any studies that investigated sex differences in nicotine seeking after a period of forced abstinence. However, sex differences in nicotine seeking have been investigated in adult rats following the extinction of nicotine-seeking behavior (Feltenstein et al., 2012). Interestingly, Feltenstein et al. (2012) did not find sex differences in cue, nicotine, or yohimbine-induced reinstatement of nicotine seeking. In contrast, in our current study, we found sex differences in nicotine seeking after a period of abstinence with no extinction training. Therefore, these findings suggest that sex differences in nicotine seeking might be observed after a period of abstinence without extinction training but not after abstinence with extinction training. Another difference between our study and the study by Feltenstein et al. (2012) where no sex differences were observed, is the strain of rats used. Feltenstein et al. (2012) used adult Sprague-Dawley rats, whereas for our study we used adult Wistar rats. Sex differences in drug seeking have been more thoroughly investigated in rats with a history of cocaine intake rather than nicotine intake. Several studies with cocaine have reported that, following forced abstinence with or without extinction training, females exhibit more cocaine seeking than males (Kerstetter et al., 2008; Nicolas et al., 2019; Corbett et al., 2021).

We also investigated the effects of nicotine and mecamylamine treatment on the self-administration of nicotine and saline. We investigated the effects of nicotine and mecamylamine on total nicotine intake over a 6-h self-administration period. Nicotine treatment did not affect operant responding for nicotine in either males or females over the 6-h self-administration period. Similarly, mecamylamine treatment did not affect nicotine self-administration in males over the same period. In a previous study, we also found that mecamylamine did not affect nicotine intake over a 6-h self-administration period in male rats (Chellian et al., 2024). In the present study, mecamylamine did, however, decrease nicotine intake in females over the 6-h self-administration period. Several studies have investigated the effects of mecamylamine on nicotine self-administration in male rats. These studies show that mecamylamine decreases nicotine intake in male rats with short or long access to nicotine (Corrigall and Coen, 1989; Paterson and Markou, 2004; DeNoble and Mele, 2006). The findings are not in line with our studies, which showed that mecamylamine did not affect nicotine intake in males over a 6-h self-administration period. This discrepancy might be due to the fact that we used a higher dose of nicotine (0.06 mg/kg/inf *versus* 0.03 mg/kg/inf) combined with a longer self-administration period (8 weeks) compared to previous studies (Corrigall and Coen, 1989; Paterson and Markou, 2004; DeNoble and Mele, 2006).

Given the short half-lives (t1/2) of nicotine (t1/2, 1 h) and mecamylamine (t1/2, 1.2 h) in rats, we also examined the time-course effects of nicotine and mecamylamine on the self-administration of nicotine and saline (Miller et al., 1977; Debruyne et al., 2003). Treatment with nicotine decreased nicotine intake at the beginning of the self-administration session. However, mecamylamine treatment increased nicotine intake during the first hour of access in the males. In a prior study, we found that nicotine decreased and mecamylamine increased first-hour nicotine intake in males with long access to nicotine (Chellian et al., 2024). These findings are in line with clinical studies demonstrating that treatment with nicotine decreases smoking and treatment with mecamylamine increases smoking (Pomerleau et al., 1987; Benowitz and Jacob, 1990; Rose et al., 2001). Interestingly, in the present study, mecamylamine treatment did not increase first-hour nicotine intake in the females. However, mecamylamine decreased nicotine intake in the females during the second and fifth hour of access. These findings indicate that mecamylamine differently affects nicotine intake in males and females. Nicotine treatment slightly decreased nicotine intake at the beginning of the session and increased intake towards the end of the session. This effect of nicotine treatment could be attributed to its locomotor depressant and stimulant effects. Previous studies have shown that nicotine treatment in drug-naïve mice and rats initially has locomotor depressant effects, followed by an increase in locomotor activity (Clarke and Kumar, 1983; Clarke, 1990; Stolerman et al., 1995; Weiss et al., 2007). Overall, our study suggests that mecamylamine has different effects in males and females.

The main goal of the present studies was to examine sex differences in nicotine intake, affective and somatic withdrawal signs, and nicotine intake after a period of forced abstinence. Overall, our findings indicate that there are significant sex differences across most of the investigated parameters related to nicotine intake, effects of mecamylamine on nicotine intake, and relapse. We discovered that female rats had higher nicotine intake during long access sessions. Furthermore, the females displayed more nicotine seeking than the males after the forced abstinence period. Mecamylamine precipitated more somatic withdrawal signs in the rats that self-administered nicotine compared to those that self-administered saline, and there was no sex difference. However, mecamylamine increased nicotine intake in males and decreased nicotine intake in females. We also found that cessation of nicotine self-administration led to spontaneous somatic withdrawal signs and an increase in locomotor activity, but no sex differences were observed in these parameters. Cessation of nicotine self-administration did not increase anxiety-like behavior or cause anhedonia in the males or the females. Furthermore, a period of forced abstinence did not lead to an increase in nicotine self-administration in the males or the females. These findings provide critical insights into the sex differences in nicotine addiction and aid in the development of more sex-specific therapeutic interventions for smoking cessation.

## Data Availability

The raw data supporting the conclusions of this article will be made available by the authors, without undue reservation.
