# Supplementary material for: Sex differences in nicotine intake and relapse behavior in nicotine-dependent adult wistar rats
Source: Front Pharmacol. 2024 Sep 26;15:1415219. doi: 10.3389/fphar.2024.1415219 (PMC11464435; doi:10.3389/fphar.2024.1415219)
Supplement: Supplementary file 1 [file DataSheet1.pdf]

Results

S1. Week 5: Baseline nicotine and saline self-administration, five 1-hour sessions

S1.1. Nicotine 0.03 mg/kg/inf and saline, three 1-hour sessions: During the first three self-administration sessions, infusions and active lever responses were higher for the rats that self-administered saline than for those that self-administered nicotine and there were no sex differences (Fig. S1A, Infusions: Sex $F_{1,46}=0.473$, NS; IVSA group $F_{1,46}=25.328$, $P < 0.001$; Sex × IVSA group $F_{1,46}=0.578$, NS; Fig. S1B, Active lever: Sex $F_{1,46}=1.18$, NS; IVSA group $F_{1,46}=26.309$, $P < 0.001$; Sex × IVSA group $F_{1,46}=0.256$, NS). Furthermore, infusions and active lever responses increased for the male and female rats that self-administered saline, but not for those that self-administered nicotine (Fig. S1A, Infusions: Days $F_{2,92} =13.885$, $P < 0.001$; Days × Sex $F_{2,92}=0.578$, NS; Days × IVSA group $F_{2,92}=15.721$, 0.001; Days × Sex × IVSA group $F_{2,92}=1.096$, NS; Fig. S1B, Active lever: Days $F_{2,92} =12.598$, $P < 0.001$; Days × Sex $F_{2,92}=0.718$, NS; Days × IVSA group $F_{2,92}=14.335$, $P < 0.001$; Days × Sex × IVSA group $F_{2,92}=1.462$, NS). Responding on the inactive lever increased over time in male and female rats that self-administered saline and nicotine (Fig. S1C, Days $F_{2,92} =10.973$, $P < 0.001$; Days × Sex $F_{2,92}=0.763$, NS; Days × IVSA group $F_{2,92}=2.206$, NS; Days × Sex × IVSA group $F_{2,92}=0.858$, NS). The rats in the saline group responded more on the inactive lever than those in the nicotine group, and the females responded more on the inactive lever than the males (Sex $F_{1,46}=6.59$, $P < 0.05$; IVSA group $F_{1,46}=23.299$, $P < 0.001$; Sex × IVSA group $F_{1,46}=2.48$, NS). Nicotine intake did not change over time and was the same in the males and the females (Fig. S1D, Days $F_{2,44} =0.454$, NS; Days × Sex $F_{2,44}=0.369$, NS; Sex $F_{1,22}=2.664$, NS).

S1.2. Nicotine 0.06 mg/kg/inf and saline, two 1-hour sessions: During the following two self-administration sessions, infusions and active lever responses were higher for the rats that self-administered saline than for those that self-administered nicotine and there were no sex differences (Fig. S1E, Infusions: Sex $F_{1,46}=1.267$, NS; IVSA group $F_{1,46}=8.135$, $P < 0.01$; Sex × IVSA group $F_{1,46}=0.153$, NS; Fig. S1F, Active lever: Sex $F_{1,46}=2.297$, NS; IVSA group $F_{1,46}=8.782$, $P < 0.01$; Sex × IVSA group $F_{1,46}=0.28$, NS). Furthermore, infusions and active lever responses increased for the male and female rats that self-administered saline, but not for those that self-administered nicotine (Fig. S1E, Infusions: Days $F_{1,46} =13.825$, $P < 0.001$; Days × Sex $F_{1,46}=0.785$, NS; Days × IVSA group $F_{1,46}=5.55$, $P < 0.05$; Days × Sex × IVSA group $F_{1,46}=0.076$, NS; Fig. S1F, Active lever: Days $F_{1,46} =12.931$, $P < 0.001$; Days × Sex $F_{1,46}=0.759$, NS; Days × IVSA group $F_{1,46}=5.089$, $P < 0.05$; Days × Sex × IVSA group $F_{1,46}=0.024$, NS). Responding on the inactive lever increased in the male and female rats that self-administered saline and nicotine (Fig. S1G, Days $F_{1,46} =15.615$, $P < 0.001$; Days × Sex $F_{1,46}=0.285$, NS; Days × IVSA group $F_{1,46}=3.767$, NS; Days × Sex × IVSA group $F_{1,46}=1.614$, NS; Sex $F_{1,46}=0.049$, NS; IVSA group $F_{1,46}=2.779$, NS; Sex × IVSA group $F_{1,46}=0.744$, NS). Nicotine intake did not change over time and was the same in the males and the females (Fig. S1H, Days $F_{1,22} =3.097$, NS; Days × Sex $F_{1,22}=0.622$, NS; Sex $F_{1,22}=0.505$, NS).

S2. Week 6 to 11: Long access nicotine and saline self-administration

Active lever: Active lever responses were higher for the rats that self-administered nicotine than for those that self-administered saline (Fig. S2A: IVSA group $F_{1,46}=62.217$, $P < 0.001$). Active lever responses were much higher for the females that self-administered nicotine than for the males that self-administered nicotine and there was also a small sex difference in the saline groups (Fig. S2A: Sex $F_{1,46}=19.005$, $P < 0.001$; Sex × IVSA group $F_{1,46}=7.01$, $P < 0.05$). The post hoc tests revealed that active lever responses were significantly higher from day 7, compared to the male nicotine group (Fig. S2A). However, on only two days (day 10 and 30) were active lever responses higher in the female saline group than in the male saline group (Fig. S2A). In addition, the post hoc tests showed that the males and females in the nicotine group had more active lever presses than the males and females in the saline group (Table S1). Active lever responses were stable in the females that self-administered nicotine but decreased in all other groups (Fig. S2A and Table S2: Days $F_{35,1610}=10.631$, $P < 0.001$; Days × Sex $F_{35,1610}=2.531$, $P < 0.001$; Days × IVSA group $F_{35,1610}=6.099$, $P < 0.001$; Days × Sex × IVSA group $F_{35,1610}=1.954$, $P < 0.001$).

Inactive lever: Inactive lever responses declined more in the rats that self-administered saline than in the rats that self-administered nicotine and stabilized at a lower level in the saline rats than in the nicotine rats (Fig. S2B and Table S2, Days $F_{35,1610}=7.782$, $P < 0.001$; Days × IVSA group $F_{35,1610}=4.091$, $P < 0.001$; IVSA group $F_{1,46}=7.779$, $P < 0.01$). There were no sex differences in responding on the inactive lever (Fig. S2B, Days × Sex $F_{35,1610}=0.882$, NS; Sex $F_{1,46}=0.614$, NS; Sex × IVSA group $F_{1,46}=0.926$, NS; Days × Sex × IVSA group $F_{35,1610}=1.158$, NS). In addition, the post hoc tests showed that males and females in the nicotine group had more inactive lever presses than the males and females in the saline group (Table S1).

S3. Long access first and last session

Active lever: Rats that self-administered nicotine had more active lever responses compared to those that self-administered saline (Fig. S3A, IVSA group $F_{1,46}=14.408$, $P < 0.001$). Additionally, female rats had more active lever responses than male rats (Fig. S3A: Sex $F_{1,46}=8.106$, $P < 0.01$; Sex × IVSA group $F_{1,46}=2.725$, NS). The number of active lever responses decreased from the first to the last session, with the greatest decrease in the saline group (Fig. S3A: Session $F_{1,46}=80.733$, $P < 0.001$; Session × Sex $F_{1,46}=0.561$, NS; Session × IVSA group $F_{1,46}=28.224$, $P < 0.001$; Session × Sex × IVSA group $F_{1,46}=0.921$, NS). The post hoc showed that both the male and the female rats in the saline group had fewer active lever responses during the last session compared to the first session (Fig. S3A).

Inactive lever: Responding on the inactive lever decreased more from the first to the last session in the saline rats than in the nicotine rats (Fig. S3B, IVSA group $F_{1,46}=0.057$, NS; Sex $F_{1,46}=0.003$, NS; Sex × IVSA group $F_{1,46}=0.107$, NS; Session $F_{1,46}=15.648$, $P < 0.001$; Session × Sex $F_{1,46}=0.081$, NS; Session × IVSA group $F_{1,46}=7.847$, $P < 0.01$; Session × Sex × IVSA group $F_{1,46}=0.317$, NS). The post hoc analysis revealed that both male and female rats in the saline group had fewer inactive lever responses during the last session compared to the first session (Fig. S3B).

S4. Withdrawal day 1: Elevated plus maze test

Nicotine abstinence did not affect the percentage of time on the open arms (Fig. S4A, IVSA group $F_{1,46}=1.103$, NS; Sex × IVSA group $F_{1,46}=0.178$, NS) and the percentage of open arm entries (Fig. S4B, IVSA group $F_{1,46}=2.334$, NS; Sex × IVSA group $F_{1,46}=0.014$, NS). Furthermore, nicotine abstinence did not affect time on the open arms (Fig. S4C, IVSA group $F_{1,46}=1.59$, NS; Sex × IVSA group $F_{1,46}=0.407$, NS), open arm entries (Fig. S4D, IVSA group $F_{1,46}=0.698$, NS; Sex × IVSA group $F_{1,46}=0.376$, NS), time in the closed arms (Fig. S4E, IVSA group $F_{1,46}=0.692$, NS; Sex × IVSA group $F_{1,46}=0.114$, NS), closed arm entries (Fig. S4F, IVSA group $F_{1,46}=0.436$, NS; Sex × IVSA group $F_{1,46}=3.665$, NS), and total distance traveled (Fig. S4G, IVSA group $F_{1,46}=1.65$, NS; Sex × IVSA group $F_{1,46}=2.646$, NS).

In comparison to the males, the females spent a higher percentage of time on the open arms (Fig. S4A, Sex $F_{1,46}=22.839$, $P < 0.001$) and had a higher percentage of open arm entries (Fig. S4B, Sex $F_{1,46}=14.064$, $P < 0.001$). The females spent more time on the open arms (Fig. S4C, Sex $F_{1,46}=22.079$, $P < 0.001$) and made more open arm entries (Fig. S4D, Sex $F_{1,46}=15.55$, $P < 0.001$) compared to the males. Additionally, the females spent less time in the closed arms (Fig. S4E, Sex $F_{1,46}=10.762$, $P < 0.01$) and traveled a greater distance (Fig. S4G, Sex $F_{1,46}=22.262$, $P < 0.001$). The number of closed arm entries was not affected by the sex of the rats (Fig. S4F, Sex $F_{1,46}=0.735$, NS). Heatmaps for both males and females in the nicotine and saline groups from the elevated plus-maze test are presented in Figure S5.

S5. Withdrawal day 2: Large open field test

Nicotine abstinence did not affect the time in the center (Fig. S6A, IVSA group $F_{1,46}=0.053$, NS; Sex × IVSA group $F_{1,46}=0.046$, NS), middle (Fig. S6B, IVSA group $F_{1,46}=0.266$, NS; Sex × IVSA group $F_{1,46}=0.252$, NS), and outside zone (Fig. S6C, IVSA group $F_{1,46}=0.119$, NS; Sex × IVSA group $F_{1,46}=0.203$, NS). The number of entries into the center zone (Fig. S6D, IVSA group $F_{1,46}=0.007$, NS; Sex × IVSA group $F_{1,46}=0.042$, NS), middle zone (Fig. S6E, IVSA group $F_{1,46}=0.357$, NS; Sex × IVSA group $F_{1,46}=0.086$, NS), and outside zone (Fig. S6F, IVSA group $F_{1,46}=0.508$, NS; Sex × IVSA group $F_{1,46}=0.111$, NS) was also unaffected by nicotine abstinence. Furthermore, the latency to enter the center zone (Fig. S6G, IVSA group $F_{1,46}=1.691$, NS; Sex × IVSA group $F_{1,46}=0.04$, NS) and middle zone (Fig. S6H, IVSA group $F_{1,46}=0.912$, NS; Sex × IVSA group $F_{1,46}=0.722$, NS) was not affected by nicotine abstinence. However, nicotine abstinence led to an increase in the distance traveled in the large open field and this parameter was not affected by the sex of the rats (Fig. S6I, IVSA group $F_{1,46}=4.283$, $P < 0.05$; Sex × IVSA group $F_{1,46}=1.656$, NS).

In the large open field test, we identified sex differences for almost all behavioral parameters. Females spent more time in the center (Fig. S6A, Sex $F_{1,46}=20.836$, $P < 0.001$) and middle (Fig. S6B, Sex $F_{1,46}=28.211$, $P < 0.001$) zones, while also making more entries into the center (Fig. S6D, Sex $F_{1,46}=45.003$, $P < 0.001$), middle (Fig. S6E, Sex $F_{1,46}=34.008$, $P < 0.001$), and outside (Fig. S6F, Sex $F_{1,46}=26.076$, $P < 0.001$) zones. In contrast, males spent more time in the outside zone (Fig. S6C, Sex $F_{1,46}=28.822$, $P < 0.001$). The females had a shorter latency to enter the center zone (Fig. S6G, Sex $F_{1,46}=18.435$, $P < 0.001$), and traveled a greater distance than the males in the large open field (Fig. S6I, Sex

$F_{1,46}=38.902$, $P < 0.001$). There was no sex differences in the latency to enter the middle zone (Fig. S6H, Sex $F_{1,46}=2.032$, NS). Heatmaps for both males and females in the nicotine and saline groups from the large open field test are presented in Figure S7.

S6. Withdrawal days 3-8: Sucrose preference test

Nicotine abstinence did not affect sucrose preference (Fig. S8, IVSA group $F_{1,43}=0.001$, NS; Sex × IVSA group $F_{1,43}=2.719$, NS). Sucrose preference was higher in the females than the males, however, sucrose preference increased over time in the males (Fig. S8, Days $F_{4,172}=4.265$, $P < 0.01$; Sex $F_{1,43}=7.64$, $P < 0.01$; Days × Sex $F_{4,172}=2.458$, $P < 0.05$; Days × IVSA group $F_{4,172}=0.131$, NS; Days × Sex × IVSA group $F_{4,172}=0.043$, NS).

S7. Week 13: Forced abstinence and nicotine intake

S7.1. Last self-administration day compared to first one after forced abstinence

Active lever: The period of abstinence led to an increase in the number of active lever presses and this increase was greater in the rats that self-administered saline than in those that self-administered nicotine (Fig. S9A: Session $F_{1,39}=22.778$, $P < 0.001$; IVSA group $F_{1,39}=21.183$, $P < 0.001$; Session × IVSA group $F_{1,39}=10.508$, $P < 0.01$). The female rats had more active lever presses than the males (Fig. S9A: Sex $F_{1,39}=7.067$, $P < 0.05$). The effect of sex on active lever presses was unaffected by the abstinence period, as well as by whether nicotine or saline was being self-administered (Fig. S9A: Session × Sex $F_{1,39}=0.218$, NS; Sex × IVSA group $F_{1,39}=2.344$, NS; Session × Sex × IVSA group $F_{1,39}=0.344$, NS).

Inactive lever: The rats that self-administered nicotine had more inactive lever responses than the rats that self-administered saline (Fig S9B, IVSA group $F_{1,39}=7.531$, $P < 0.01$). Inactive lever responses were not affected by the sex of the rats or the abstinence period (Fig. S9B, Session $F_{1,39}=3.701$, NS; Sex $F_{1,39}=0.299$, NS; Session × Sex $F_{1,39}=2.944$, NS; Session × IVSA group $F_{1,39}=0.039$, NS; Sex × IVSA group $F_{1,39}=0$, NS; Session × Sex × IVSA group $F_{1,39}=2.688$, NS).

S7.2. Self-administration after forced abstinence, six self-administration sessions

Active lever: The females had more active lever responses than the males (Fig. S10A: Sex $F_{1,39}=14.45$, $P < 0.001$). Furthermore, active lever responses were higher for the rats that self-administered nicotine than for those that self-administered saline (Fig. S10A: IVSA group $F_{1,39}=33.127$, $P < 0.01$; Sex × IVSA group $F_{1,39}=0.3$, NS). Active lever responses decreased over time, and this effect was greatest in the males that self-administered saline (Fig. S10A: Days $F_{5,195}=25.715$, $P < 0.001$; Days × Sex $F_{5,195}=1.847$, NS; Days × IVSA group $F_{5,195}=4.156$, $P < 0.001$; Days × Sex × IVSA group $F_{5,195}=2.359$, $P < 0.05$). The post hoc tests showed that active lever responses were higher in the nicotine group than in the saline group (Fig. S10A). Furthermore, females in both the nicotine and saline group had more active lever responses compared to the males (Fig. S10A).

Inactive lever: Inactive lever responses decreased over time and this was not affected by the sex of the rats (Fig. S10B, Days $F_{5,195} = 5.664$, $P < 0.001$; Days × Sex $F_{5,195} = 0.383$, NS; Days × IVSA group $F_{5,195} = 0.95$, NS; Days × Sex × IVSA group $F_{5,195} = 0.133$, NS). Furthermore, rats that self-administered saline had fewer inactive lever responses than the rats that self-administered nicotine (Fig. S10B, Sex $F_{1,39} = 1.031$, NS; IVSA group $F_{1,39} = 5.192$, $P < 0.05$; Sex × IVSA group $F_{1,39} = 0.694$, NS).

S8. Week 14: Mecamylamine treatment and self-administration

Active lever: Active lever presses were higher in females than in males, and they were higher in rats that self-administered nicotine compared to those that self-administered saline (Fig S11A: Sex $F_{1,39} = 6.457$, $P < 0.05$; IVSA group $F_{1,39} = 29.66$, $P < 0.001$). Mecamylamine decreased the number of active lever responding in the females that self-administered nicotine. However, it increased the active lever responses in the females that self-administered saline, while not affecting active lever responses in the males (Fig S11A: Mecamylamine treatment $F_{1,39} = 0.177$, NS; Mecamylamine treatment × Sex $F_{1,39} = 1.044$, NS; Mecamylamine treatment × IVSA group $F_{1,39} = 2.164$, NS; Sex × IVSA group $F_{1,39} = 0.217$, NS; Mecamylamine treatment × Sex × IVSA group $F_{1,39} = 9.728$, $P < 0.01$). The post hoc analysis showed that infusions and active lever presses were higher in mecamylamine-treated females that self-administered saline than in saline-treated females that self-administered saline (Fig S11A).

Inactive lever: Mecamylamine treatment increased inactive lever responses and this parameter was not affected by the sex of the rats (Fig S11B, Mecamylamine treatment $F_{1,39} = 13.821$, $P < 0.001$; Mecamylamine treatment × Sex $F_{1,39} = 1.707$, NS; Mecamylamine treatment × IVSA group $F_{1,39} = 0.625$, NS; Sex × IVSA group $F_{1,39} = 0.079$, NS; Mecamylamine treatment × Sex × IVSA group $F_{1,39} = 0.03$, NS; Sex $F_{1,39} = 0.056$, NS). Furthermore, the rats that self-administered nicotine had more inactive lever responses than those that self-administered saline (IVSA group $F_{1,39} = 8.068$, $P < 0.01$).

S9. Week 15: Nicotine treatment and self-administration

Active lever: The administration of nicotine ten minutes before the self-administration sessions did not affect the number active lever responses (Fig. S11C: Nicotine treatment $F_{1,39} = 0.107$, NS; Nicotine treatment × Sex $F_{1,39} = 0.307$, NS; Nicotine treatment × IVSA group $F_{1,39} = 2.279$, NS; Nicotine treatment × Sex × IVSA group $F_{1,39} = 0.002$, NS). The female rats had more active lever presses compared to the males (Fig. S11C, Active lever: Sex $F_{1,39} = 9.098$, $P < 0.01$). Furthermore, the rats that self-administered nicotine had more active lever responses than the rats that self-administered saline and this was not affected by the sex of the rats (Fig. S11C: IVSA group $F_{1,39} = 13.863$, $P < 0.001$; Sex × IVSA group $F_{1,39} = 0.003$, NS).

Inactive lever: Treatment with nicotine or the sex of the rats did not affect inactive lever responses (Fig. S11D, Nicotine treatment $F_{1,39} = 0.711$, NS; Sex $F_{1,39} = 1.908$, NS; Nicotine treatment × Sex $F_{1,39} = 1.939$, NS; Nicotine treatment × IVSA group $F_{1,39} = 0.088$, NS; Sex × IVSA group $F_{1,39} = 2.067$, NS; Nicotine treatment × Sex × IVSA group $F_{1,39} = 1.216$,

NS). However, the rats that self-administered nicotine had more inactive lever responses than the rats that self-administered saline (Fig. S11D, IVSA group $F_{1,39}=15.698$, $P < 0.001$).

S10. Week 18: Forced abstinence and nicotine- and saline-seeking behavior - Last self-administration day compared to seeking session after forced abstinence

Active lever: The rats that self-administered nicotine had more active lever responses than the rats that self-administered saline (Fig. S12A: IVSA group $F_{1,38}=42.745$, $P < 0.001$). Following a period of abstinence, active lever presses increased, and this effect was greater in the rats with a history of nicotine self-administration than in the rats with a history of saline self-administration (Fig. S12A: Session $F_{1, 38} = 121.197$, $P < 0.001$; Session × IVSA group $F_{1,38} =13.959$, $P < 0.001$). Additionally, the females had more active lever presses than the males (Fig. S12A: Sex $F_{1,38}=20.6$, $P < 0.001$; Session × Sex $F_{1, 38} = 3.727$, NS; Sex × IVSA group $F_{1,38}=2.093$, NS; Session × Sex × IVSA group $F_{1,38} =2.336$, NS).

Inactive lever: The rats that self-administered nicotine had more responses on the inactive lever than the rats that self-administered saline (Fig. S12B: IVSA group $F_{1,38}=9.655$, $P < 0.01$). Furthermore, inactive lever presses increased after abstinence (Fig. S12B: Session $F_{1, 38} = 19.172$, $P < 0.001$; Session × IVSA group $F_{1,38} =2.967$, NS). There was a strong trend towards a greater abstinence-induced increase in inactive lever responses in the males than the females (Fig. S12B: Sex $F_{1,38}=0.243$, NS; Session × Sex $F_{1, 38} = 3.889$, $P=0.056$; Sex × IVSA group $F_{1,38}=1.051$, NS; Session × Sex × IVSA group $F_{1,38} =0.04$, NS).

Tables

Table S1: Week 6 to 11, nicotine and saline self-administration in male and female rats for 36 days (saline vs nicotine group).

Days	Saline IVSA vs nicotine IVSA (0.06 mg/kg/inf)							
	Fig. 2A: Infusions_ 6 h IVSA		Fig. S2A: Active lever responses_6 h IVSA		Fig. S2B: Inactive lever responses_6 h IVSA		Fig. 2B: Infusions_1 h IVSA	
	males	females	males	females	males	females	males	females
1	ns	ns	ns	ns	ns	ns	ns	ns
2	ns	ns	ns	ns	ns	ns	ns	ns
3	***	ns	***	ns	ns	ns	ns	ns
4	***	***	***	**	ns	ns	ns	*
5	***	***	**	***	ns	ns	ns	**
6	***	***	**	***	ns	ns	**	*
7	**	***	**	***	ns	ns	ns	**
8	**	***	**	***	ns	ns	ns	**
9	*	***	*	***	ns	ns	ns	**
10	**	***	***	***	ns	ns	ns	***
11	**	***	**	***	ns	ns	**	***
12	***	***	***	***	ns	ns	**	***
13	***	***	***	***	ns	ns	***	***
14	***	***	***	***	ns	ns	**	***
15	**	***	**	***	ns	ns	**	***
16	***	***	***	***	ns	ns	***	***
17	***	***	**	***	ns	ns	**	***
18	***	***	***	***	ns	ns	***	***
19	***	**	***	***	ns	ns	***	***
20	*	***	*	***	ns	ns	ns	***
21	***	***	***	***	ns	ns	*	***
22	***	***	***	***	ns	ns	ns	***
23	**	***	***	***	ns	ns	**	***
24	***	***	***	***	*	ns	***	***
25	***	***	***	***	ns	ns	***	***
26	***	***	***	***	ns	ns	***	***
27	***	***	***	***	ns	ns	***	***
28	**	***	**	***	ns	**	*	***
29	***	***	***	***	ns	ns	*	***
30	***	***	***	***	ns	ns	**	***
31	***	***	***	***	ns	ns	***	***
32	***	***	***	***	ns	ns	***	***
33	***	***	***	***	ns	ns	***	***
34	***	***	***	***	ns	ns	***	***
35	**	***	**	***	ns	ns	***	***
36	***	***	***	***	ns	*	**	***

Bonferroni's multiple comparisons post hoc test: Asterisks indicate more infusions, active lever responses, and inactive levers responses in the nicotine group than in the saline group of same sex and at the same day. Saline IVSA-male (N=14) vs nicotine IVSA-male (N=12) and saline IVSA-female (N=12) vs nicotine IVSA-female (N=12). * $P < 0.05$, ** $P < 0.01$, *** $P < 0.001$.

Table S2: Week 6 to 11, nicotine and saline self-administration in male and female rats for 36 days (compared to day 1).

Days	Six-hour IVSA: compared to day 1 IVSA												First hour IVSA: compared to day 1 IVSA			
	Fig. 2A: Infusions				Fig. S2A: Active lever responses				Fig. S2B: Inactive lever responses				Fig. 2B: Infusions			
	S-M	S-F	N-M	N-F	S-M	S-F	N-M	N-F	S-M	S-F	N-M	N-F	S-M	S-F	N-M	N-F
2	***	***	ns	ns	***	***	ns	ns	ns	***	ns	ns	***	***	ns	ns
3	***	***	ns	ns	***	***	ns	ns	***	***	ns	ns	***	***	ns	ns
4	***	***	ns	ns	***	***	ns	ns	***	***	ns	ns	***	***	ns	ns
5	***	***	*	ns	***	***	*	ns	***	***	ns	ns	***	***	ns	ns
6	***	***	ns	ns	***	***	ns	ns	***	***	ns	*	***	***	ns	ns
7	***	***	**	ns	***	***	**	ns	***	***	ns	*	***	***	ns	ns
8	***	***	**	ns	***	***	**	ns	***	***	*	*	***	***	ns	ns
9	***	***	***	ns	***	***	**	ns	***	***	ns	*	***	***	*	ns
10	**	**	***	ns	***	***	**	ns	***	**	ns	ns	**	**	ns	***
11	***	***	**	*	***	***	*	*	***	***	ns	ns	***	***	ns	***
12	***	***	**	ns	***	***	ns	ns	***	***	ns	*	***	***	ns	**
13	***	***	ns	ns	***	***	ns	ns	***	***	ns	ns	***	***	ns	**
14	***	***	ns	*	***	***	ns	ns	***	***	ns	ns	***	***	ns	***
15	***	***	**	ns	***	***	*	ns	***	***	ns	ns	***	***	ns	*
16	***	***	*	ns	***	***	*	ns	***	***	ns	ns	***	***	ns	**
17	***	***	ns	ns	***	***	ns	ns	***	***	ns	ns	***	***	ns	**
18	***	***	ns	ns	***	***	ns	ns	***	***	ns	ns	***	***	ns	ns
19	***	***	ns	ns	***	***	ns	ns	***	***	ns	ns	***	***	ns	ns
20	***	***	ns	ns	***	***	ns	ns	***	***	ns	ns	***	***	ns	ns
21	***	***	ns	ns	***	***	ns	ns	***	***	ns	ns	***	***	ns	ns
22	***	***	ns	ns	***	***	ns	ns	***	***	ns	ns	***	***	ns	ns
23	***	***	**	ns	***	***	**	ns	***	***	*	ns	***	***	ns	ns
24	***	***	*	ns	***	***	ns	ns	***	***	ns	ns	***	***	ns	**
25	***	***	ns	ns	***	***	ns	ns	***	***	ns	ns	***	***	ns	**
26	***	***	**	ns	***	***	*	ns	***	***	ns	ns	***	***	ns	ns
27	***	***	**	ns	***	***	*	ns	***	***	ns	ns	***	***	ns	ns
28	***	***	**	ns	***	***	**	ns	***	***	ns	ns	***	***	ns	**
29	***	***	**	ns	***	***	*	ns	***	***	ns	ns	***	***	ns	ns
30	***	***	***	ns	***	***	**	ns	***	***	ns	ns	***	***	ns	***
31	***	***	ns	ns	***	***	ns	ns	***	***	ns	ns	***	***	ns	ns
32	***	***	*	ns	***	***	ns	ns	***	***	ns	ns	***	***	ns	ns
33	***	***	*	ns	***	***	*	ns	***	***	ns	ns	***	***	ns	ns
34	***	***	**	ns	***	***	**	ns	***	***	ns	ns	***	***	ns	ns
35	***	***	**	ns	***	***	**	ns	***	***	ns	ns	***	***	ns	ns
36	***	***	**	ns	***	***	*	ns	***	***	ns	ns	***	***	ns	ns

Bonferroni's multiple comparisons post hoc test: Asterisks indicate a significant difference from day 1 in rats of the same sex and the same self-administration group. * $P < 0.05$, ** $P < 0.01$, *** $P < 0.001$. Abbreviations and group size: S-M, saline IVSA-male (N=14); N-M, nicotine IVSA-male (N=12); S-F, saline IVSA-female (N=12); N-F, nicotine IVSA-female (N=12).

Figures

Figure S1

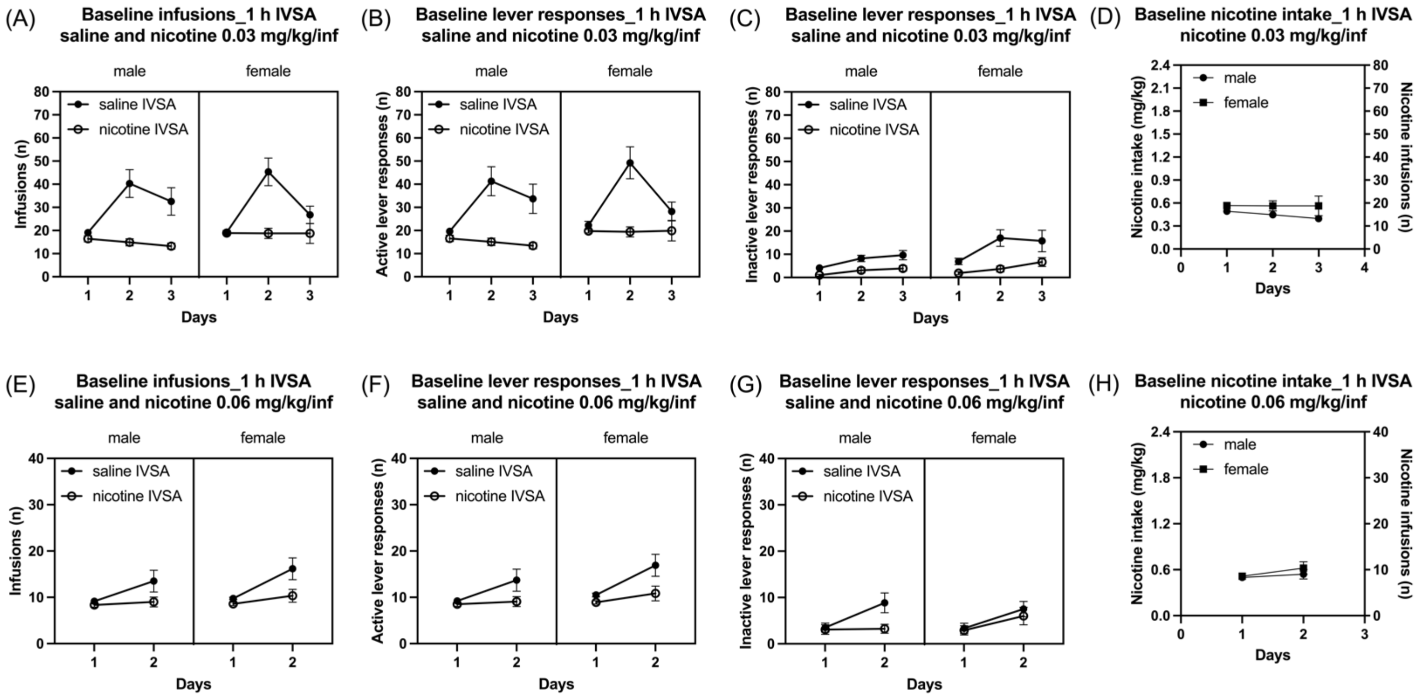

Figure S1. Baseline nicotine and saline self-administration in male and female rats. At the onset of the self-administration study, the rats self-administered 0.03 mg/kg/inf of nicotine and saline for three days (A, infusions; B, active lever responses; C, inactive lever responses; D, nicotine intake) and then 0.06 mg/kg/inf of nicotine and saline for two days in 1 h sessions (E, infusions; F, active lever responses; G, inactive lever responses; H, nicotine intake). During the first three sessions (0.03 mg/kg/inf of nicotine), infusions and responding on the active lever were higher in the saline group than in the nicotine group (A, B). Inactive lever responses were higher in the saline group than in the nicotine group (C). Nicotine intake was the same in males and females (D). During the following two sessions (0.06 mg/kg/inf of nicotine), infusions and active lever responses were higher for the rats that self-administered saline than for those that self-administered nicotine (E, F). Responding on the inactive lever increased over time in both the saline and nicotine group (G). Nicotine intake was the same in males and females (H). Saline IVSA-male (N=14), nicotine IVSA-male (N=12), saline IVSA-female (N=12), and nicotine IVSA-female (N=12). Data are expressed as means ± SEM.

Figure S2

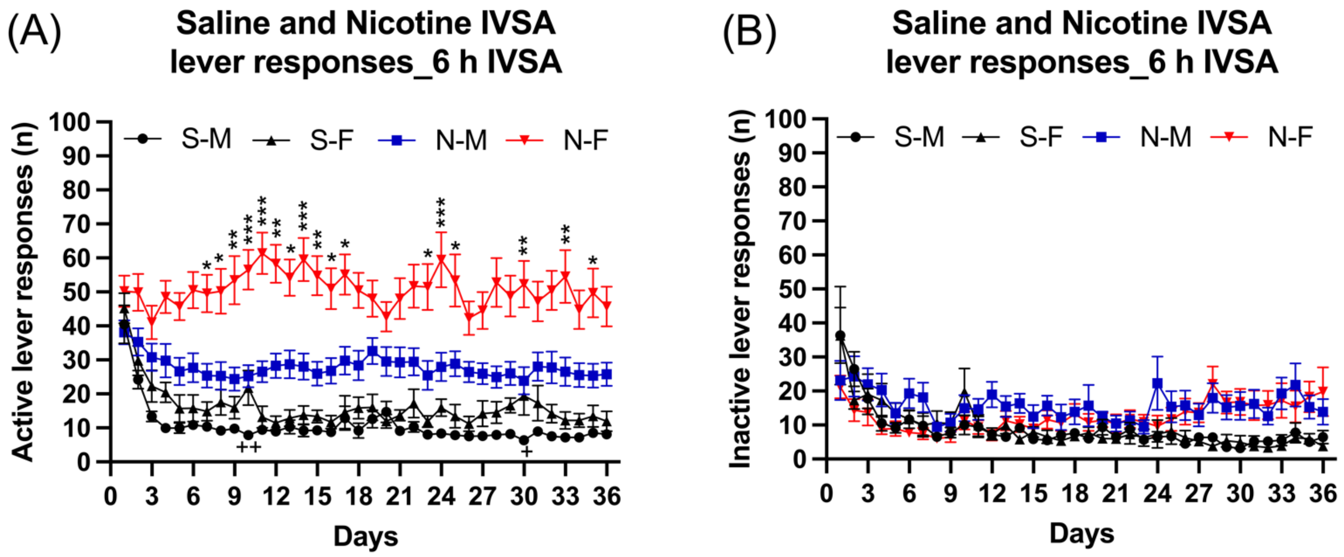

Figure S2. Operant responding for nicotine and saline during daily six-hour self-administration sessions in male and female rats. The rats self-administered nicotine (0.06 mg/kg/inf) and saline in 6-hour self-administration sessions for 36 days. Active lever responses (A) and inactive lever responses (B) in 6-hour nicotine and saline self-administration sessions. Asterisks indicate more active lever responses in the females that self-administered nicotine than in the males that self-administered nicotine on the same test day. Plus signs indicate more active lever responses in the females that self-administered saline than in the males that self-administered saline on the same test day. *, + $P<0.05$; **, ++ $P<0.01$; *** $P<0.001$. Abbreviations and group size: S-M, saline IVSA-male (N=14); N-M, nicotine IVSA-male (N=12); S-F, saline IVSA- female (N=12); N-F, nicotine IVSA-female (N=12). Data are expressed as means ± SEM.

Figure S3

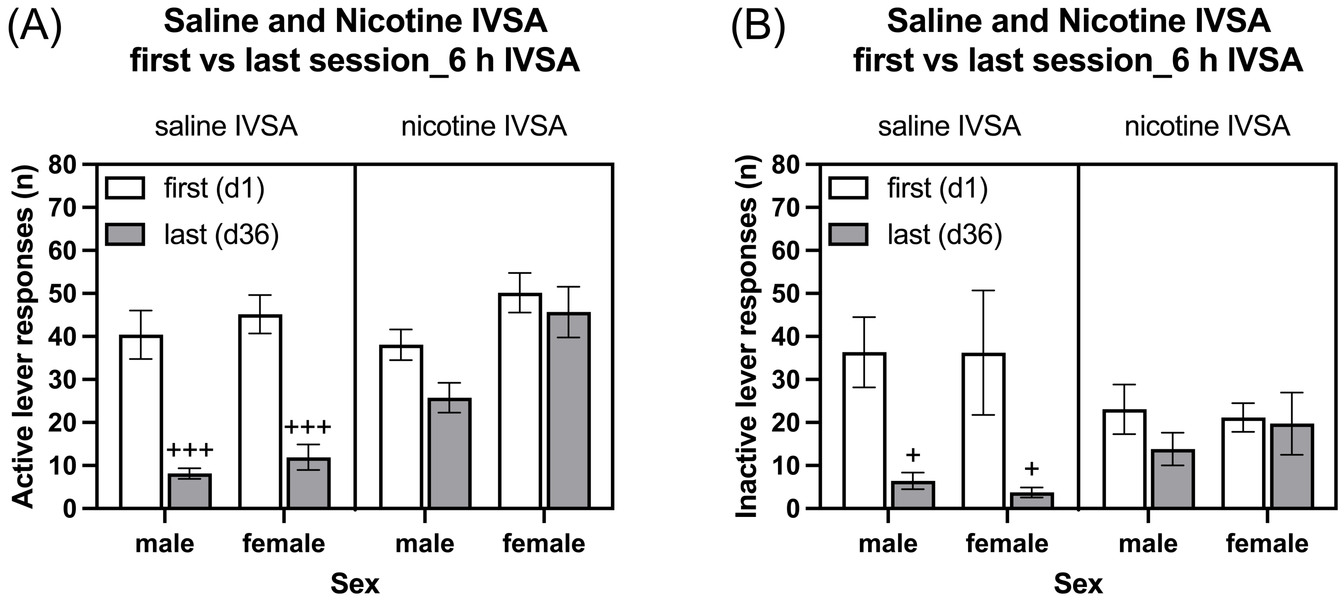

Figure S3. Operant responding for nicotine and saline during the first and last self-administration session in male and female rats. The rats self-administered saline and nicotine 0.06 mg/kg/inf in 6-hour self-administration sessions for 36 days and responses during the first (day 1) and last (day 36) self-administration session are shown. Active lever responses (A) and inactive lever responses (B) in 6-hour nicotine and saline self-administration sessions. Plus signs indicate a significant decrease in active and inactive lever responses in the last session compared to the first session with the same self-administration group and same sex. +, $P<0.05$; +++, $P<0.001$. Saline IVSA-male (N=14), nicotine IVSA-male (N=12), saline IVSA- female (N=12), and nicotine IVSA-female (N=12). Data are expressed as means ± SEM.

Figure S4

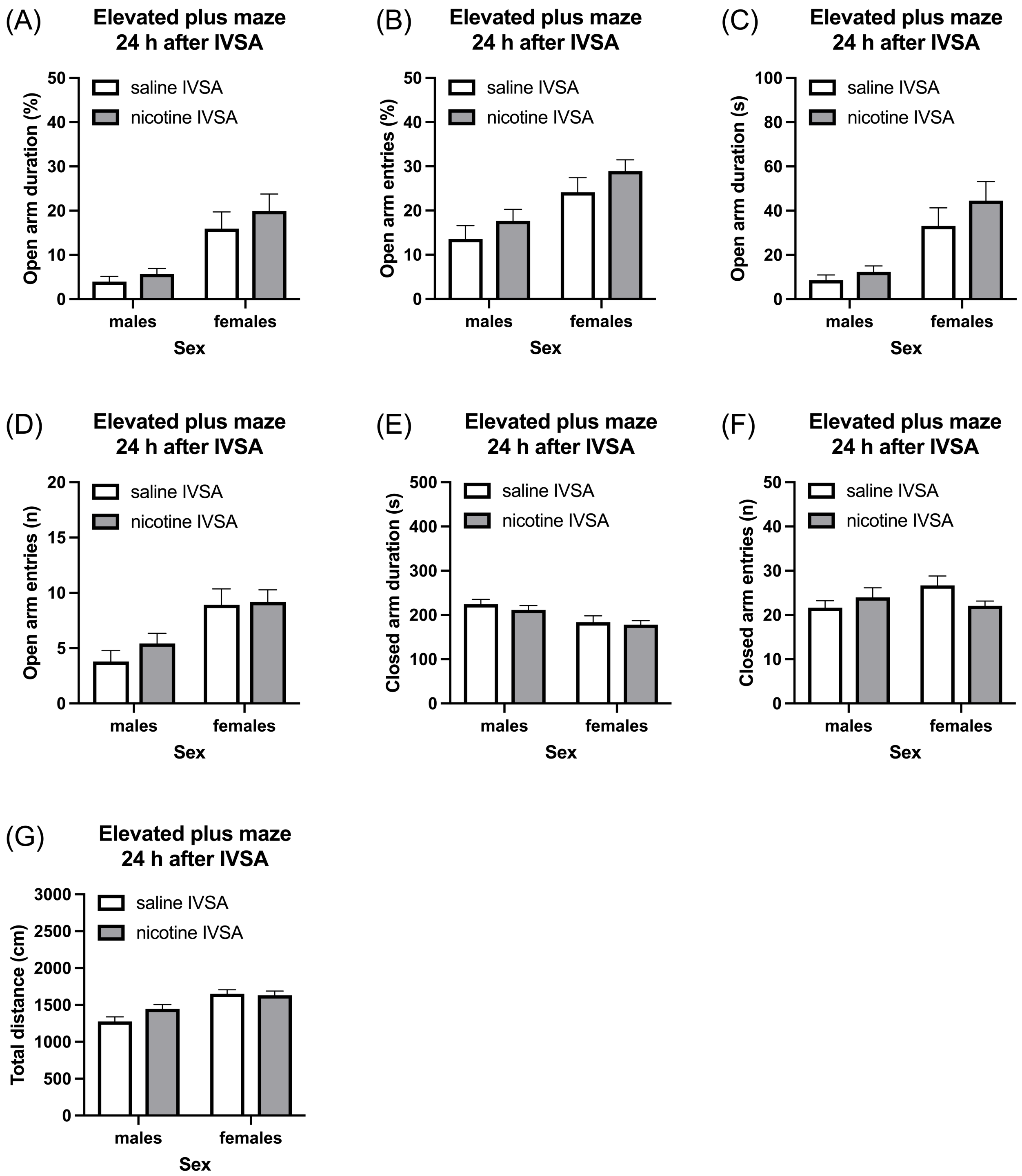

Figure S4. Nicotine withdrawal and anxiety like behavior in the elevated plus maze test in male and female rats. Nicotine withdrawal did not affect behavior (A-G) in the elevated plus maze test in male and female rats. Saline IVSA-male (N=14), nicotine IVSA-male (N=12), saline IVSA- female (N=12), and nicotine IVSA-female (N=12). Data are expressed as means ± SEM.

Figure S5

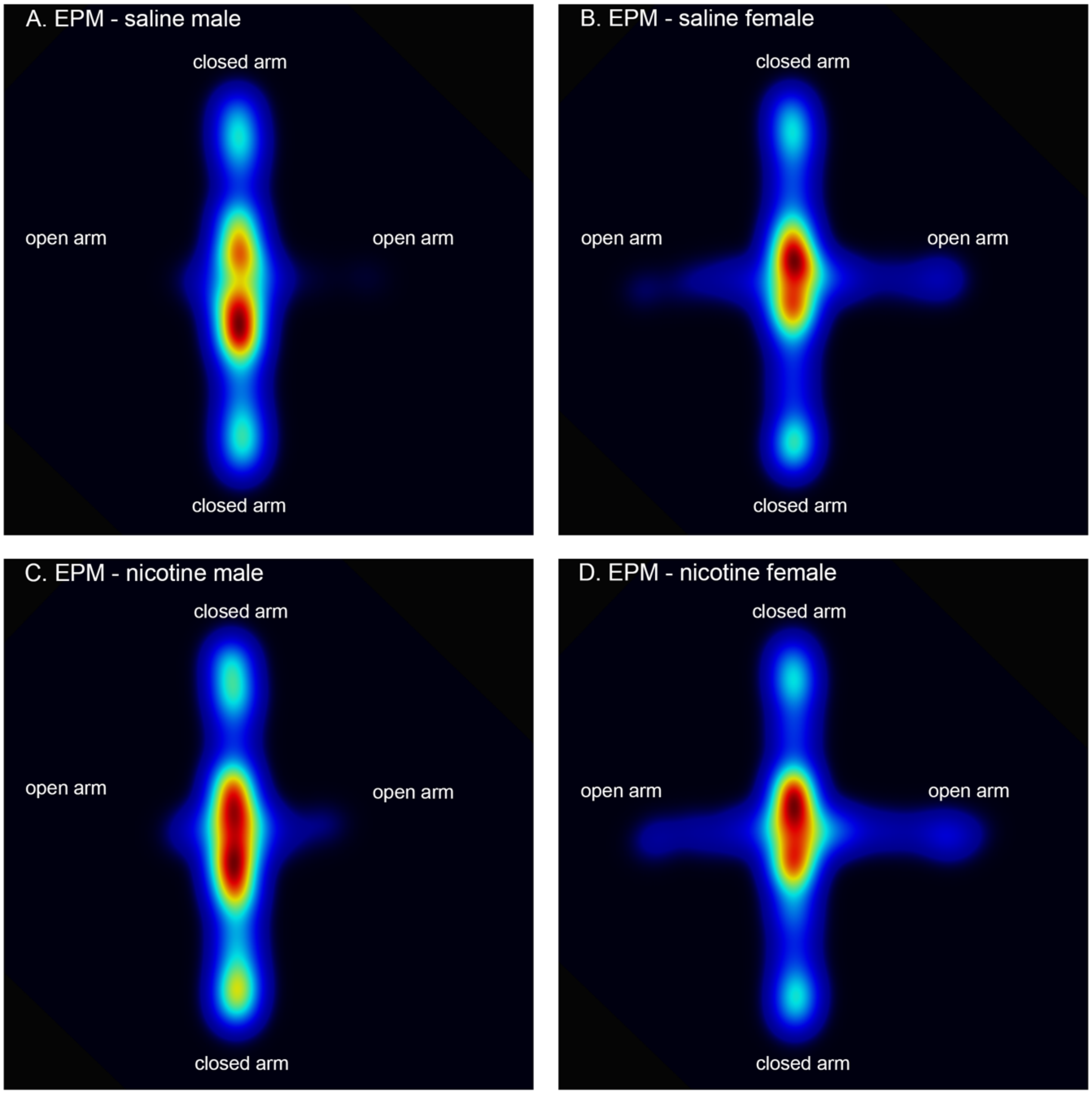

Figure S5. Heatmaps of the effects of nicotine withdrawal and sex in the elevated plus-maze test. The figures show that nicotine withdrawal did not affect behavior in the elevated plus maze test in male and female rats. The females spent more time on the open arms than the males (A-D). The figures were generated with EthoVision software, and warmer colors indicate more time spent in a specific area. Saline IVSA-male (N=14), nicotine IVSA-male (N=12), saline IVSA- female (N=12), and nicotine IVSA-female (N=12). Abbreviations: EPM, elevated plus-maze.

Figure S6

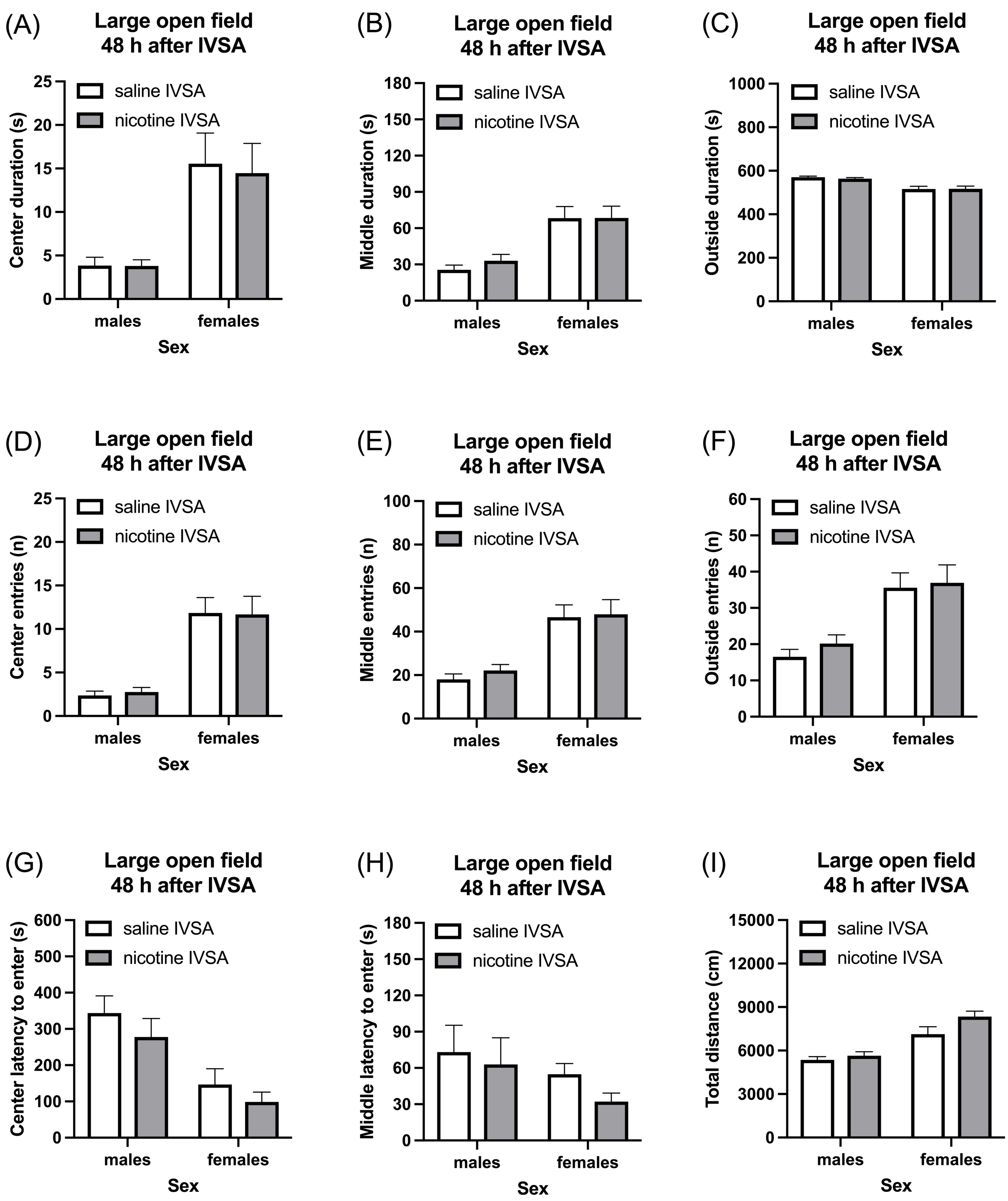

Figure S6. Nicotine withdrawal and anxiety like behavior in the large open field test in male and female rats. Nicotine withdrawal did not affect behavior (A-I) in the large open field test in male and female rats. Saline IVSA-male (N=14), nicotine IVSA-male (N=12), saline IVSA- female (N=12), and nicotine IVSA-female (N=12). Data are expressed as means ± SEM.

Figure S7

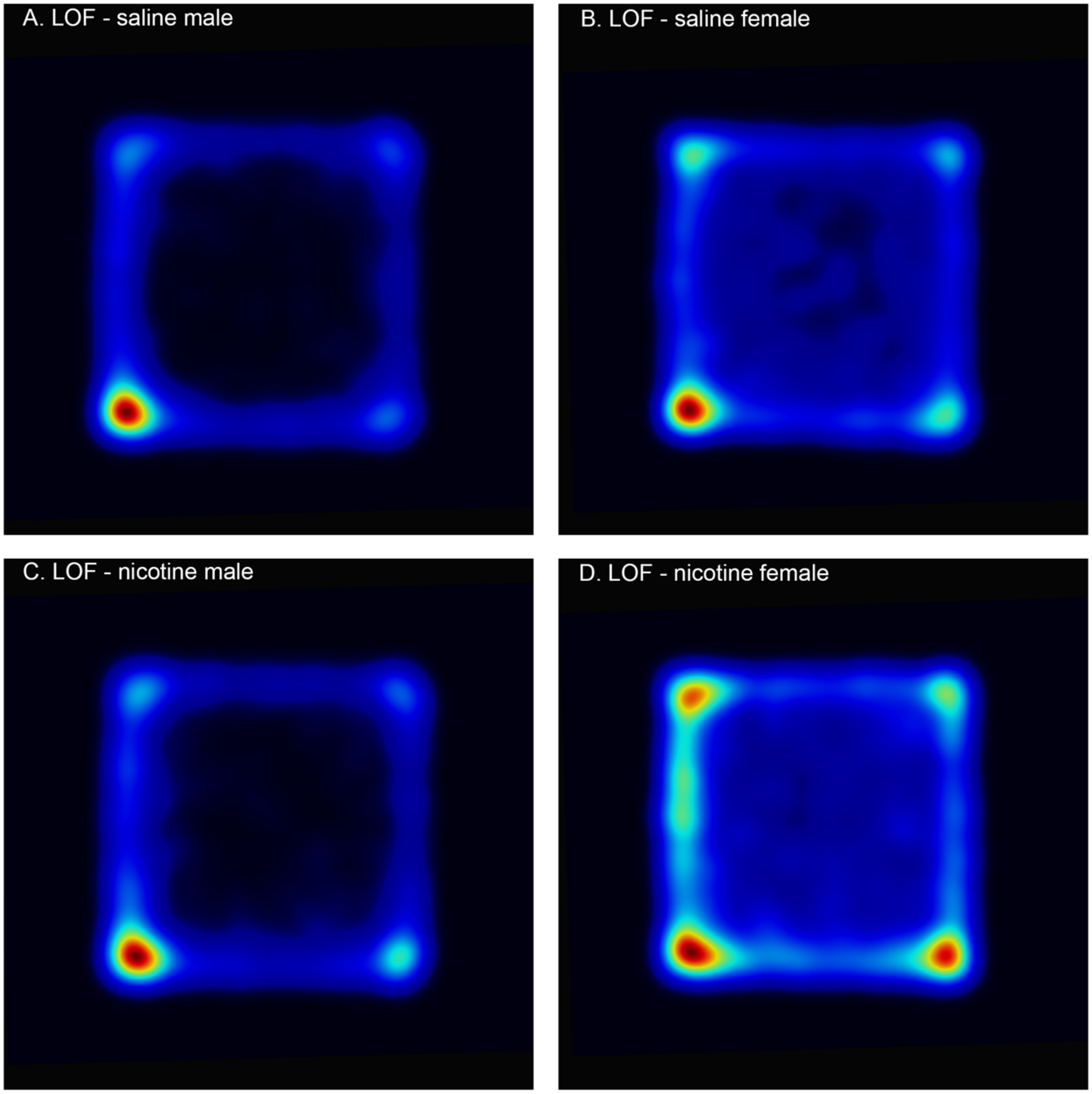

Figure S7. Heatmaps of the effects of nicotine withdrawal and sex in the large open field test. The figures show that nicotine withdrawal did not affect behavior in the large open field test in male and female rats. The females spent more time in the center than the males (A-D). The figures were generated with EthoVision software, and warmer colors indicate more time spent in a specific area. Saline IVSA-male (N=14), nicotine IVSA-male (N=12), saline IVSA- female (N=12), and nicotine IVSA-female (N=12). Abbreviations: LOF, large open field.

Figure S8

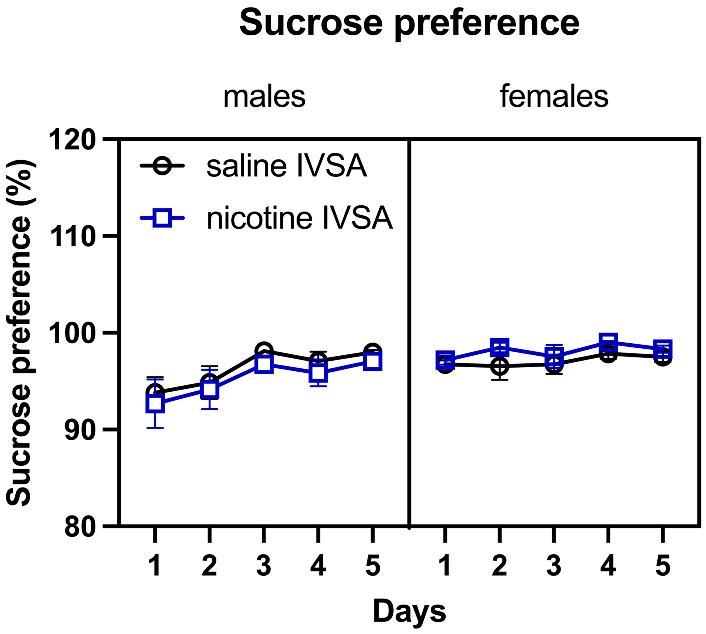

Figure S8. Nicotine withdrawal and sucrose preference in male and female rats. Nicotine withdrawal did not affect sucrose preference in male and female rats. Saline IVSA-male (N=13), nicotine IVSA-male (N=12), saline IVSA- female (N=11), and nicotine IVSA-female (N=11). Data are expressed as means ± SEM.

Figure S9

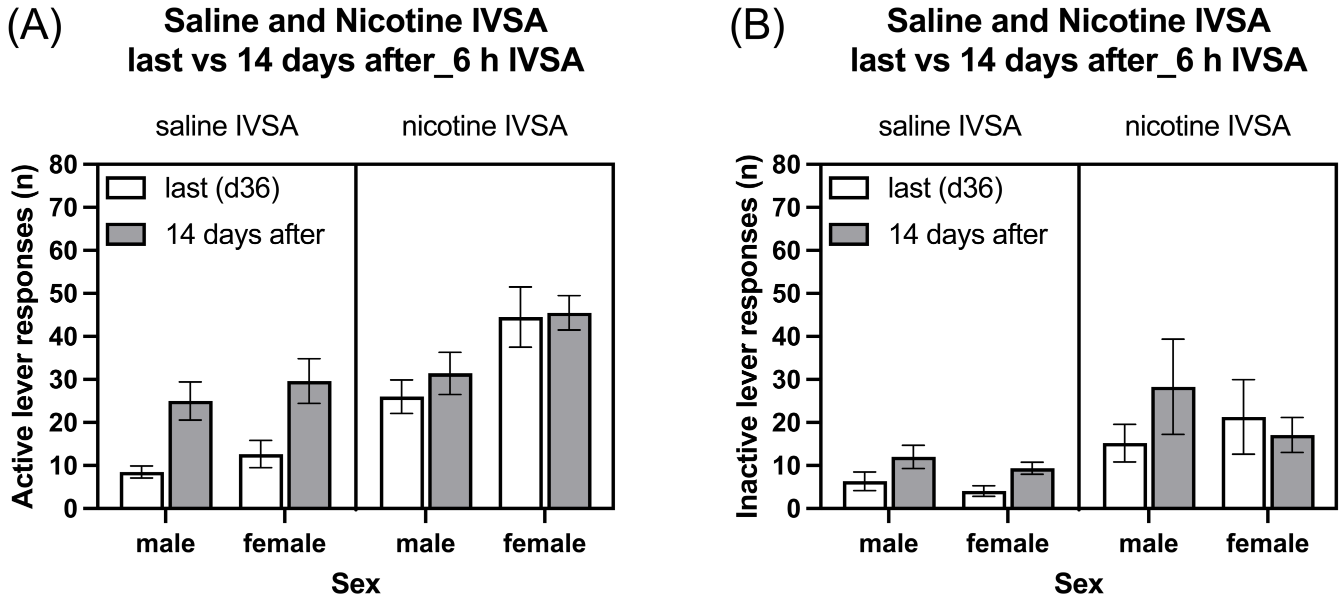

Figure S9. Operant responding for nicotine and saline immediately after forced abstinence in male and female rats. Fourteen days after last self-administration (day 36), the rats self-administered saline and nicotine 0.06 mg/kg/inf in 6-hour self-administration sessions. Active lever responses (A) and inactive lever responses (B) in in 6-hour nicotine and saline self-administration sessions. Saline IVSA-male (N=12); nicotine IVSA-male (N=10); saline IVSA- female (N=11); nicotine IVSA-female (N=10). Data are expressed as means ± SEM.

Figure S10

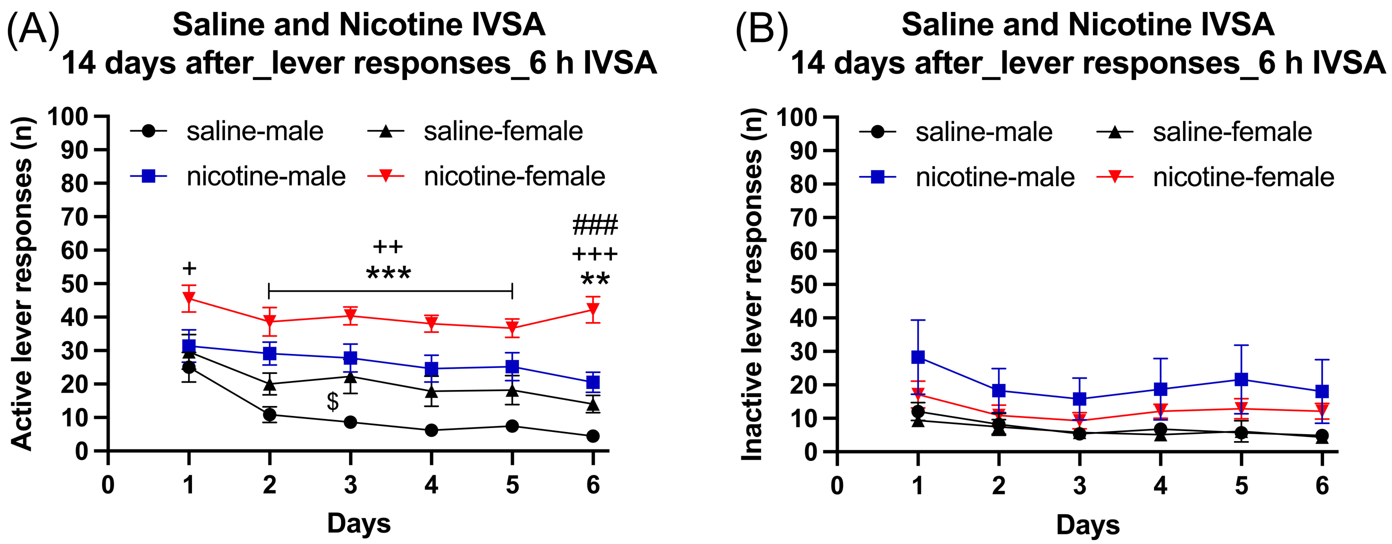

Figure S10. Daily operant responding for nicotine and saline after forced abstinence in male and female rats. After a 14-day abstinence period, the rats self-administered saline and 0.06 mg/kg/inf of nicotine in 6-hour self-administration sessions for 6 days. Active lever responses (A), and inactive lever responses (B) in 6-hour nicotine and saline self-administration sessions. Asterisks indicate more responses on the active lever in the nicotine group males than in the saline group males on the same day (A). Plus signs indicate more responding on the active lever in the nicotine group females than in the saline group females at the same day (A). Dollar signs indicate more responding on the active lever responses in the saline group females than in the saline group males on the same day (A). Pound sign indicate more active lever responses in females than in males that self-administered nicotine on the same day (A). +, $ $P<0.05$; **, ++ $P<0.01$; ***, +++, ### $P<0.001$. Saline IVSA-male (N=12); nicotine IVSA-male (N=10); saline IVSA- female (N=11); nicotine IVSA-female (N=10). Data are expressed as means ± SEM.

Figure S11

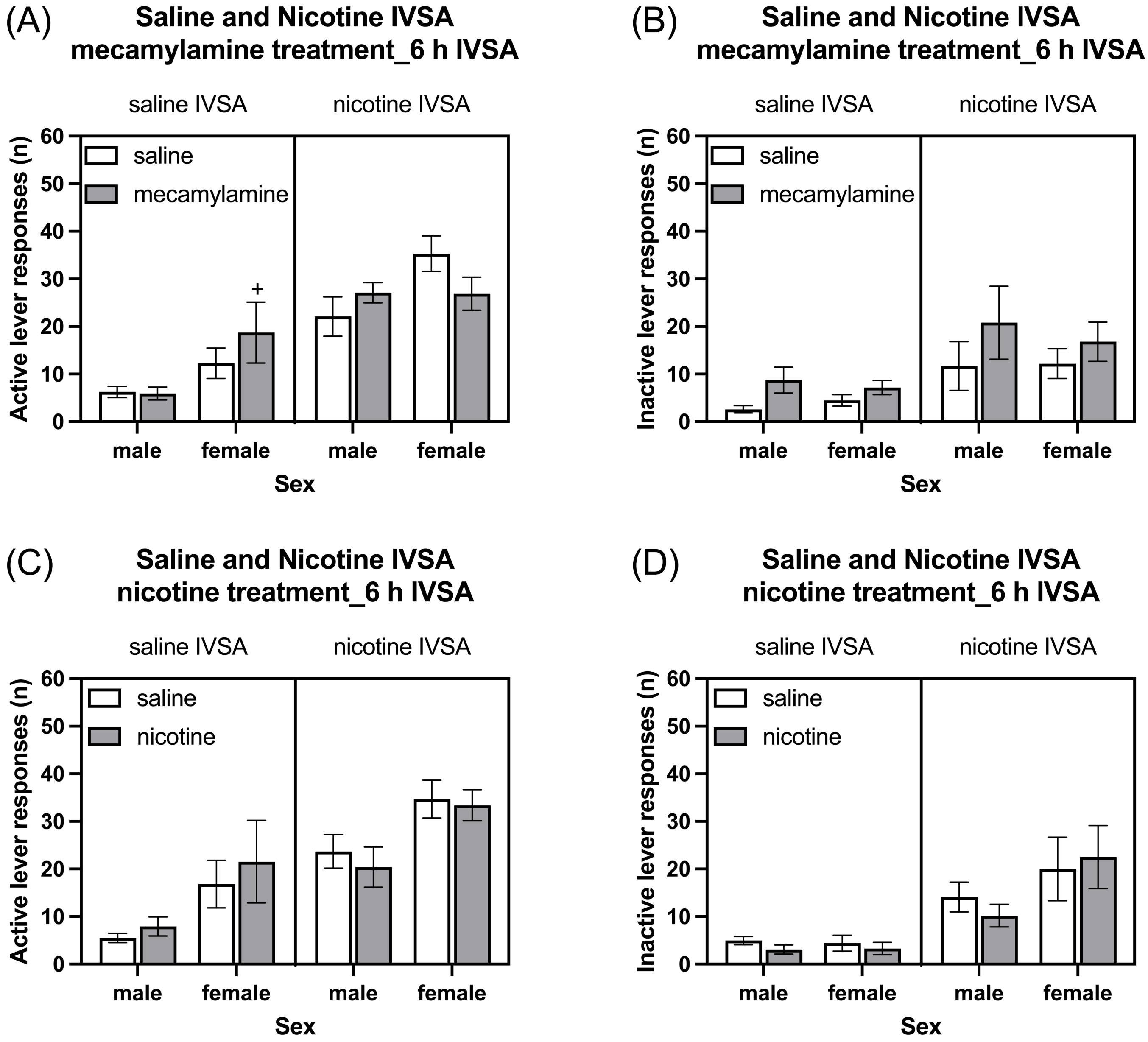

Figure S11. Effects of mecamylamine and nicotine treatment on operant responding for nicotine and saline in male and female rats. Active lever responses (A) and inactive lever responses (B) in 6-hour nicotine and saline self-administration sessions after mecamylamine treatment. Active lever responses (C) and inactive lever responses (D) in 6-hour nicotine and saline self-administration sessions after nicotine treatment. The plus sign indicate more active lever responses in rats treated with mecamylamine compared to those treated with the vehicle, within the same self-administration group and sex. + $P<0.05$. Saline IVSA-male (N=12), nicotine IVSA-male (N=10), saline IVSA- female (N=11), and nicotine IVSA-female (N=10). Data are expressed as means ± SEM.

Figure S12

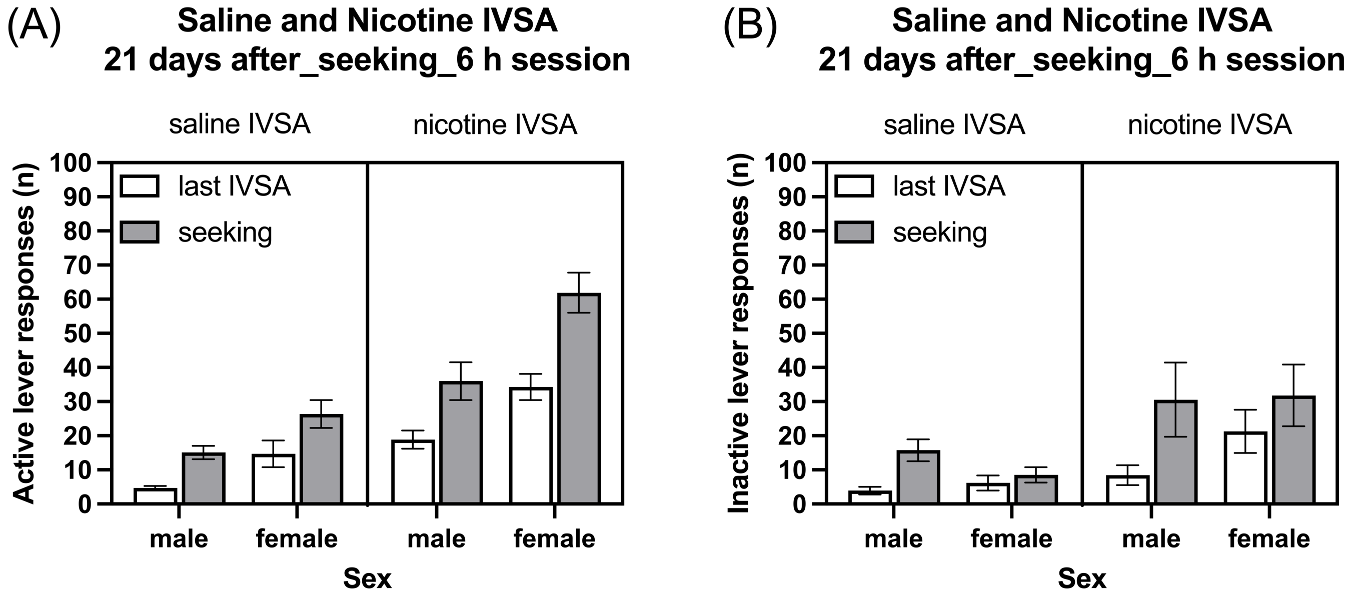

Figure S12. Active and inactive lever presses indicating nicotine and saline seeking behavior after forced abstinence in male and female rats. Twenty-one days after last self-administration session, the rats were placed in the operant chamber and the lever responses were recorded in a 6-hour session. Active lever responses (A) and Inactive lever responses (B) in 6-hour sessions. Saline IVSA-male (N=12); nicotine IVSA-male (N=9); saline IVSA- female (N=11); nicotine IVSA-female (N=10). Data are expressed as means ± SEM.
